# Tenomodulin promotes human adipocyte differentiation and beneficial visceral adipose tissue expansion

**DOI:** 10.1038/ncomms10686

**Published:** 2016-02-16

**Authors:** Ozlem Senol-Cosar, Rachel J. Roth Flach, Marina DiStefano, Anil Chawla, Sarah Nicoloro, Juerg Straubhaar, Olga T. Hardy, Hye Lim Noh, Jason K. Kim, Martin Wabitsch, Philipp E. Scherer, Michael P. Czech

**Affiliations:** 1 Program in Molecular Medicine, University of Massachusetts Medical School, Worcester, Massachusetts 01605, USA; 2 Department of Internal Medicine, Touchstone Diabetes Center, The University of Texas Southwestern Medical Center, Dallas, Texas 75390, USA; 3 Department of Medicine, University of Massachusetts Medical School, Worcester, Massachusetts 01605, USA; 4 Division of Pediatric Endocrinology and Diabetes, Department of Pediatrics and Adolescent Medicine, University Medical Center Ulm, Ulm 89075, Germany

## Abstract

Proper regulation of energy storage in adipose tissue is crucial for maintaining insulin sensitivity and molecules contributing to this process have not been fully revealed. Here we show that type II transmembrane protein tenomodulin (*TNMD*) is upregulated in adipose tissue of insulin-resistant versus insulin-sensitive individuals, who were matched for body mass index (BMI). *TNMD* expression increases in human preadipocytes during differentiation, whereas silencing *TNMD* blocks adipogenesis. Upon high-fat diet feeding, transgenic mice overexpressing *Tnmd* develop increased epididymal white adipose tissue (eWAT) mass, and preadipocytes derived from *Tnmd* transgenic mice display greater proliferation, consistent with elevated adipogenesis. In *Tnmd* transgenic mice, lipogenic genes are upregulated in eWAT, as is *Ucp1* in brown fat, while liver triglyceride accumulation is attenuated. Despite expanded eWAT, transgenic animals display improved systemic insulin sensitivity, decreased collagen deposition and inflammation in eWAT, and increased insulin stimulation of Akt phosphorylation. Our data suggest that TNMD acts as a protective factor in visceral adipose tissue to alleviate insulin resistance in obesity.

## Abstract (web summary)

Expansion of visceral adipose tissue is usually associated with insulin resistance and metabolic disease. Here, the authors show that the membrane protein TNMD is upregulated in visceral fat of insulin resistant obese individuals and promotes healthy adipose tissue expansion through increasing adipogenesis.

## Graphical abstract

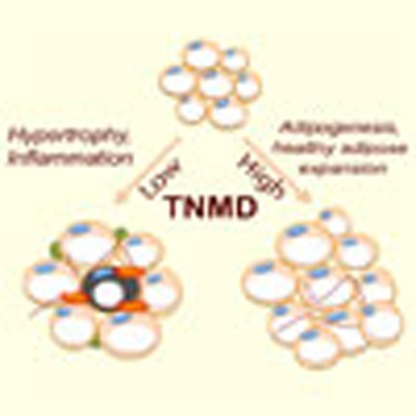

A large body of work has suggested that adipose tissue plays a key role in determining metabolic health as a major regulator of carbohydrate and lipid homeostasis. Expansion of adipose tissue in overweight or obese humans can lead to a spectrum of dysfunctions collectively referred to as metabolic syndrome. However, a significant number of metabolically healthy obese human subjects demonstrate a situation of benign adipose tissue expansion whose differences from pathological obesity are poorly understood^1^,^2^,^3^,^4^,^5^. Some studies have suggested that specific physiological mechanisms and anatomical locations of adipose expansion may differentially affect metabolic homeostasis^6^,^7^,^8^,^9^. Major white adipose depots located in subcutaneous regions and the visceral cavity can dynamically expand during obesity^10^. In humans, adipose tissue expands via adipocyte hypertrophy during early obesity, whereas an increase in adipocyte number, denoted hyperplasia, also occurs in prolonged obesity^11^,^12^. Animal models have demonstrated that subcutaneous adipose tissue enlargement is mostly due to hypertrophy, while the visceral depot expands by increasing both cell size and number upon long-term high-fat diet (HFD) feeding^13^,^14^. This increase in cell number derives from the differentiation of adipocyte precursors into differentiated adipocytes, a well-defined process that has been extensively modelled in the 3T3-L1 mouse cell line^15^,^16^. Though mouse adipocyte lines such as 3T3-L1 cells have greatly contributed to identifying the molecular mechanisms involved in differentiation and maintaining mature adipocyte function^17^, interspecies differences in gene expression and regulation between mouse and human adipocytes are important to consider and further investigate^18^,^19^.

Central obesity is linked to several metabolic morbidities such as type 2 diabetes and cardiovascular disease^20^. Visceral adipose tissue is more prone to inflammation than subcutaneous fat in obesity through mechanisms that enhance immune cell content^21^ and increase pro-inflammatory cytokine expression^22^,^23^,^24^,^25^. A leading hypothesis suggests that low-grade inflammation in fat depots is involved in metabolic syndrome^26^,^27^. Moreover, visceral adipose tissue may be more lipolytic than subcutaneous adipose tissue due to dampened insulin suppression of lipolysis and a higher response to catecholamines. This, in turn, increases both non-esterified fatty acid release into the circulation and hepatic lipid deposition due to the close proximity of visceral adipose tissue to the hepatic portal vein^28^,^29^. Ectopic lipid storage in the liver and muscle is thought to trigger insulin resistance in these tissues, although not under all conditions^30^. Therefore, promoting healthy expansion and better lipid storage in visceral adipose tissue is crucial to maintain glucose homeostasis and insulin sensitivity.

To identify and explore mechanisms in adipose tissues that either cause insulin resistance or preserve insulin sensitivity in obese individuals, we compared gene expression in subcutaneous and omental adipose tissues from obese human subjects matched for body mass index (BMI) but differing in insulin resistance. Among several differentially expressed genes identified, we focused on tenomodulin (*TNMD*), a type II transmembrane protein, due to its high and predominant expression in human adipose tissue, also noted by others^31^,^32^. Comparative analysis of adipose tissue *TNMD* expression in obese and lean individuals also previously indicated that TNMD is strongly correlated with BMI^31^,^33^,^34^. Moreover, many genome-wide association studies revealed that single-nucleotide polymorphisms in the *TNMD* gene are associated with various metabolic characteristics such as BMI, serum low-density lipoprotein levels and inflammatory factors^35^,^36^,^37^. Though these studies indicate a potential role for TNMD in human adipose tissue, the function of TNMD has not been evaluated.

Here by gene silencing and generating a transgenic mouse line, we demonstrate that TNMD is required for adipocyte differentiation, and overexpression of *Tnmd* in adipose tissue protects mice from obesity-induced systemic insulin resistance. These data suggest that adipocyte TNMD is a protective factor that enhances insulin sensitivity in obesity, potentially via promoting hyperplasia and beneficial lipid storage in the visceral adipose tissue.

## Results

### Higher expression of human *TNMD* in insulin resistance

**Figure 1:**
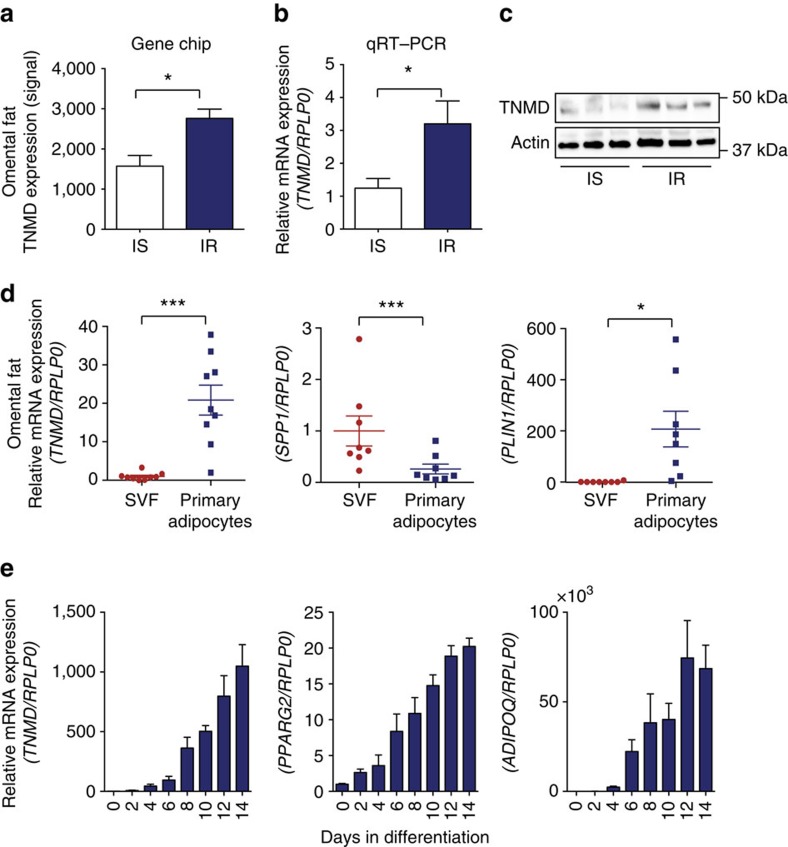
Adipose tissue expression of *TNMD* in obese humans and adipocytes. Omental adipose tissue was isolated from obese patients undergoing bariatric surgery. (**a**) RNA was isolated, and a microarray was performed (mean±s.e.m.; *n*=6, insulin sensitive; *n*=8, insulin resistant, *P<0.05, by Student's *t*-test). (**b**) Quantitative PCR validation of *TNMD* expression in obese individuals (mean±s.e.m.; *n*=6, insulin sensitive; *n*=8, insulin resistant, *P<0.05, by Student's *t*-test). (**c**) Western blot analysis of TNMD and actin in omental adipose tissue lysates from insulin-sensitive and insulin-resistant patients (*n*=4–5, *P*=0.023 by Student's *t*-test). (**d**) Quantitative PCR analysis of *TNMD*, *PLIN1* and *SPP1* expression in the stromal vascular fraction (SVF) and in primary adipocytes isolated from omental adipose tissue of obese individuals (mean±s.e.m.; *n*=9 for both SVF and primary adipocytes *P<0.05, **P<0.01, ***P<0.001, by Student's *t*-test). (**e**) Quantitative PCR analysis of *TNMD*, *PPARG2* and *ADIPOQ* expression at various time points during differentiation in SGBS human adipocytes (mean±s.e.m.; *n*=3–4, *P<0.05, **P<0.01, ***P<0.001, by Student's *t*-test).

To assess gene expression differences in insulin-resistant versus insulin-sensitive obese individuals, total RNA was isolated from snap-frozen adipose tissue biopsies from omental and subcutaneous fat depots of human subjects undergoing bariatric surgery, and analysed for genome-wide gene expressions. Clinical data of the subjects used in gene expression analysis are shown in Supplementary Table 1. DNA microarray data identified *TNMD* as a gene that is significantly upregulated in omental fat from insulin-resistant individuals compared with BMI-matched, insulin-sensitive subjects (Fig. 1a). However, no significant difference in *TNMD* gene expression was observed between the two groups in subcutaneous adipose tissue (Supplementary Fig. 1a). These results were validated by assessing messenger RNA (mRNA) and protein levels using quantitative PCR with reverse transcription (qRT–PCR) and western blotting, respectively (Fig. 1b,c). It was previously demonstrated that white adipose tissue and primary adipocytes have the highest expression of *TNMD* among human tissues^31^. Although this original study claimed similar *TNMD* expression in the stromal vascular fraction (SVF) and whole adipose tissue, we observed that *TNMD* is predominantly expressed in adipocytes compared with cells from the SVF in both visceral and subcutaneous white adipose tissues (Fig. 1d; Supplementary Fig. 1b).

To assess the role of TNMD in human adipocyte function, we utilized human preadipocytes obtained from a subject with Simpson–Golabi–Behmel syndrome (SGBS cells)^38^ and discovered that *TNMD* expression increased by several hundred fold during adipocyte differentiation in culture (Fig. 1e). Expression of mature adipocyte markers such as *PPARG2* and *ADIPOQ* certified that these cells had undergone adipocyte differentiation after induction with the adipogenic cocktail. *TNMD* expression was increased even 18 h after stimulation of the preadipocytes. Moreover, *TNMD* exhibited a time-dependent expression profile similar to peroxisome proliferator-activated receptor gamma 2 (*PPARG2*) and CCAAT/enhancer-binding protein alpha (*C/EBPA*), but not Kruppel-like factor 4 (*KLF4*), which is an early transcription factor in the adipogenic cascade (Supplementary Fig. 1c).

### TNMD is required for human adipogenesis

**Figure 2:**
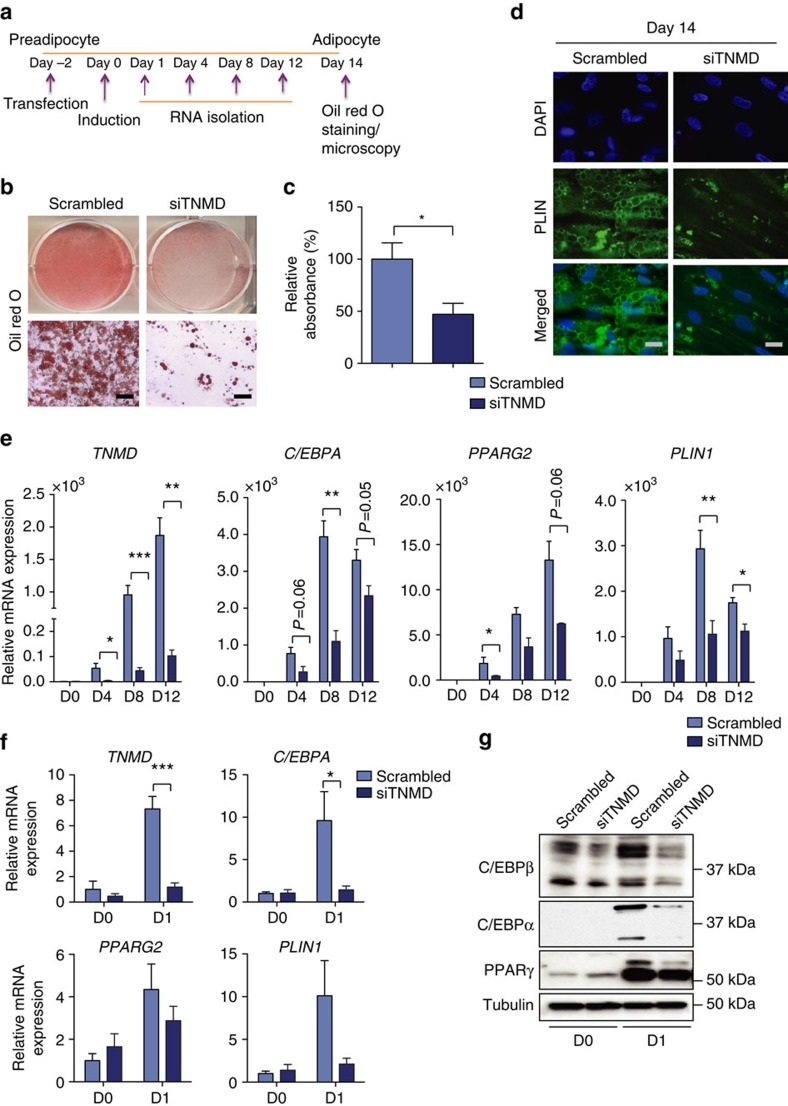
siRNA-mediated silencing of *TNMD* attenuates differentiation in human adipocytes. SGBS preadipocytes were transfected with siRNA 2 days before adipogenesis induction. Total RNA isolation and Oil Red O staining were performed on the stated days of differentiation. (**a**) Schematic demonstration of experimental method. (**b**,**c**) Adipocytes were differentiated for 14 days in culture and stained with Oil Red O. (**b**) Representative images (scale bar, 100 μm). (**c**) Quantification of Oil Red O staining (mean±s.e.m.; *n*=4 *P<0.05, Student's *t*-test). (**d**) Immunofluorescent staining of mature adipocytes with PLIN (green) and 4,6-diamidino-2-phenylindole (DAPI; blue; scale bar, 20 μm). (**e**) Quantitative PCR analysis of *TNMD*, *C/EBPA*, *PPARG2* and *PLIN1* expression at days 0, 4, 8 and 12 of adipocyte differentiation in SGBS cells (mean±s.e.m.; *n*=3 *P<0.05, **P<0.01, ***P<0.001). (**f**) Quantitative PCR analysis of *TNMD*, *C/EBPA*, *PPARG2* and *PLIN1* expression at day 0 and day 1 after stimulation (mean±s.e.m.; *n*=3 *P<0.05, **P<0.01, ***P<0.001, by Student's *t*-test). (**g**) Immunoblots for C/EBPβ, C/EBPα, PPARγ and tubulin at day 0 and day 1 of adipogenic differentiation (*n*=3 for C/EBPβ and PPARγ, *n*=6 for C/EBPα, quantified in Supplementary Fig. 2 by Student's *t*-test).

Several studies have demonstrated that adipose tissue expansion in obesity is promoted by adipogenesis in addition to adipocyte hypertrophy in both mice and humans^14^,^39^,^40^,^41^,^42^. Because *TNMD* had an expression profile that was similar to genes that are critically involved in adipocyte differentiation and its expression is increased in obese adipose tissue, we hypothesized that TNMD might be involved in human adipocyte differentiation. To test this hypothesis, *TNMD* was silenced in SGBS preadipocytes two days before adipogenic stimulation (Fig. 2a). Remarkably, silencing of *TNMD* significantly attenuated differentiation as demonstrated by substantially decreased accumulation of neutral lipids measured by Oil Red O staining at day 14 (Fig. 2b,c). Analysis of mature SGBS adipocytes also revealed that preadipocytes lacking TNMD had fewer lipid droplets and lacked a differentiated adipocyte morphology as assessed by perilipin (PLIN) staining (Fig. 2d). Thus, TNMD is required for human adipocyte differentiation.

Unlike the mouse cell line 3T3-L1, human primary adipocytes do not undergo clonal expansion^43^,^44^. SGBS cells also did not display any increase in cell number during the first 5 days of differentiation (data not shown), suggesting that clonal expansion does not occur in these cells. Therefore, full confluence is important for SGBS cells to differentiate. To assess whether TNMD affected cell death in SGBS preadipocytes, MTT (3-(4,5-dimethylthiazolyl-2)-2,5-diphenyltetrazolium bromide) cell viability assay was performed to assess viability in *TNMD*-silenced SGBS cells. The results show short interfering RNA (siRNA)-mediated silencing of *TNMD* did not affect viability (Supplementary Fig. 2a).

To further investigate whether TNMD is required for adipogenic gene expression during differentiation, total mRNA was isolated at different time points of differentiation (Fig. 2a) and expression of adipogenic markers such as *PLIN1*, *C/EBPA* and *PPARG2* were analysed by qRT–PCR. Indeed, expression of these genes was significantly blunted at most of the time points in *TNMD*-silenced cells compared with cells that had been transfected with scrambled siRNA (Fig. 2e). Because the expression of adipogenic transcription factors increases markedly even during the first day of differentiation, we asked whether *TNMD* silencing diminishes the early induction of these transcription factors as well as their target genes. Thus, a DNA microarray analysis was performed in SGBS cells that had been transfected with either scrambled or TNMD siRNA at day 1 of differentiation. The induction of many adipogenic genes was significantly diminished after TNMD depletion (Supplementary Fig. 2b). Importantly, in the absence of TNMD, stimulation of both the early transcription factor CCAAT/enhancer-binding protein beta (C/EBPβ) as well as late transcription factors C/EBPα and peroxisome proliferator-activated receptor gamma (PPARγ) was diminished (Fig. 2f,g; Supplementary Fig. 2c). Although *TNMD* expression is low in murine adipocytes, TNMD depletion in 3T3-L1 preadipocytes revealed that TNMD is also required for mouse adipocyte differentiation and plays a similar role to regulate adipogenic gene expression as in SGBS cells (Supplementary Fig. 2d,e). These data suggest that even though expression profiles are different between mature human and mouse adipocytes, the requirement of TNMD for differentiation is conserved between the two species.

The C-terminal domain of TNMD is similar to the secreted and functional portion of its homologous protein chondromodulin 1 (ref. 32), and studies have suggested that the C-terminal domain of TNMD is functionally active^45^. We sought to understand whether the effect of TNMD to regulate adipogenesis is cell autonomous. Thus, SGBS cells treated with siTNMD were mixed with non-transfected cells in equal numbers to determine whether wild-type TNMD rescues the adipogenic defect in TNMD-silenced cells via a paracrine mechanism. After adipogenic stimulation, we observed that Oil Red O staining was diminished by ∼50% when control cells were co-cultured with transfected cells, suggesting that the adipogenic effect of TNMD is cell autonomous *in vitro* (Supplementary Fig. 3a).

### Increased eWAT expansion in *Tnmd* overexpressing mice

**Figure 3:**
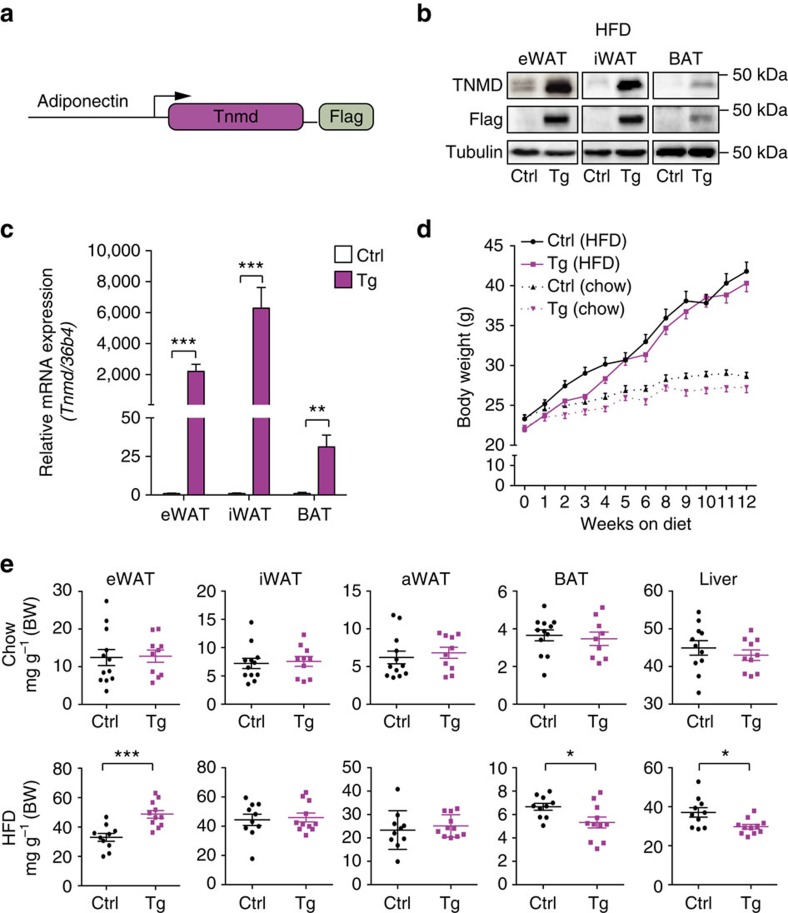
Specific *Tnmd* expression in adipose tissue enhances HFD-induced epididymal fat expansion. (**a**) Schematic of transgene construct used to generate adipose tissue-specific *Tnmd* transgenic mice. (**b**) Representative TNMD, Flag and tubulin immunoblots in iWAT, eWAT and BAT lysates that were isolated from male control (Ctrl) and transgenic mice (Tg) fed a HFD for 16 weeks. Same samples were run on different gels for TNMD. (**c**) Quantitative PCR analysis of *Tnmd* overexpression in adipose tissues from both chow- and HFD-fed male animals. (**d**) Six-week-old male control (Ctrl) and transgenic (Tg) mice were fed chow or HFD for 12 weeks. Body weights were measured at the indicated time points (HFD: *n*=11 (control), *n*=11 (transgenic); chow: *n*=11 (control), *n*=7 (transgenic)). (**e**) Epididymal, inguinal and axillary white adipose tissue, brown adipose tissue and liver weights were measured in control (Ctrl) or transgenic (Tg) male mice after 16 weeks of chow or HFD and normalized to overall body weight (mean±s.e.m.; chow: *n*=12 (control), *n*=9 (transgenic); HFD: *n*=10 (control), *n*=11 (transgenic); *P<0.05, **P<0.01, ***P<0.001, by Student's *t*-test).

To gain a better understanding of the role of *Tnmd* in adipose tissue, we generated transgenic mice that overexpress mouse *Tnmd* under the adipose tissue-specific Adiponectin promoter (Fig. 3a). Two transgenic lines (hereafter, line 1 and line 2) that exhibited significant overexpression of *Tnmd* in inguinal white adipose tissue (iWAT), epididymal white adipose tissue (eWAT) and brown adipose tissue (BAT) were utilized in these experiments (Fig. 3b,c). qRT–PCR and western blot analysis demonstrated that *Tnmd* expression was specific to adipose tissue in these mice (Supplementary Fig. 4a,b). Given that *TNMD* expression is increased during obesity in human adipose tissue^31^, weight gain was assessed in the *Tnmd* transgenic mice. In both transgenic lines, no weight difference was observed compared with littermate controls after both chow and HFD-fed conditions (Fig. 3d; Supplementary Fig. 4c). However, a significant increase in eWAT weight was observed in HFD-fed but not in chow-fed transgenic animals (Fig. 3e). The transgenic mice also displayed a concomitant decrease in liver weight after HFD, suggesting that the *Tnmd* transgenic mice may have enhanced adipose tissue storage capacity, which may attenuate lipid deposition in non-adipose tissues. Transgenic mice also had significantly smaller BAT when compared with controls (Fig. 3e). However, no significant difference was detected in inguinal and axillary white adipose tissue weights after either feeding regimens (Fig. 3e).

### TNMD promotes healthy visceral adipose tissue expansion

**Figure 4:**
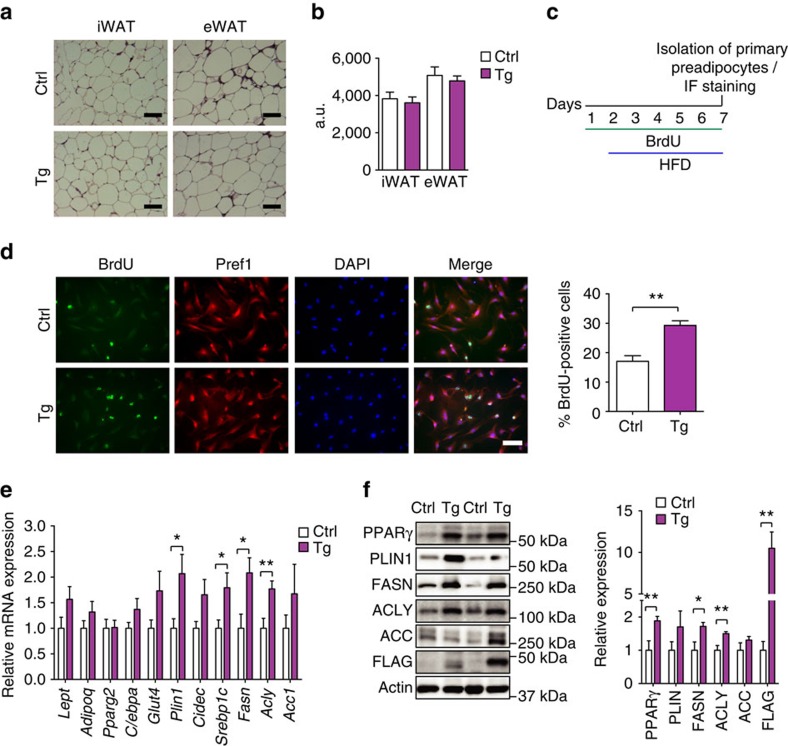
Healthy visceral adipose tissue expansion in *Tnmd* transgenic mice. Six-week-old male control (Ctrl) and *Tnmd* transgenic (Tg) animals were fed chow or HFD for 16 weeks as indicated. (**a**) Representative haematoxylin and eosin images of eWAT and iWAT (*n*=5 (control), *n*=9 (transgenic); scale bar, 100 μm). (**b**) Adipocyte size was analysed using Adiposoft software. At least four different areas per mouse were analysed, and the average adipocyte size in each group was calculated (mean±s.e.m.; *n*=5 (control), *n*=9 (transgenic); Student's *t*-test). (**c**) Experimental set-up for *in vivo* BrdU labelling in primary preadipocytes. Six-week-old female mice were treated with 0.8 mg ml^−1^ BrdU in water containing 1% sucrose. (**d**) Preadipocytes were isolated, seeded on coverslips and stained with BrdU and Pref1 antibodies. At least three different areas were quantified in each slide, and the percentage of BrdU-positive cells were calculated (mean±s.e.m.; *n*=5 for both group; *P<0.05, **P<0.01 by Student's *t*-test). Scale bar, 50 μm. (**e**) Quantitative PCR and (**f**) western blot analysis of adipogenic and lipogenic genes in eWAT of HFD-fed male animals (mean±s.e.m.; *n*=9 (control), *n*=11 (transgenic) (qRT–PCR); *n*=5 (control), *n*=9 (transgenic) (western blot); *P<0.05, **P<0.01 by Student's *t*-test).

Because *Tnmd* transgenic mice had larger eWAT (Fig. 3e) and because TNMD is required for adipogenesis (Fig. 2), white adipose tissue cell size was assessed to understand whether the significant increase in eWAT weight in *Tnmd* transgenic mice was due to hypertrophy or hyperplasia. Though the eWAT pads were larger, no significant difference in adipocyte size was observed in eWAT or iWAT depots of HFD-fed *Tnmd* transgenic mice (Fig. 4a,b), and this was also the case after a short-term (4 weeks) HFD (Supplementary Fig. 5). These results suggest that the increased eWAT weight (Fig. 3e) was caused by an increase in adipocyte number rather than hypertrophy.

We thus investigated whether preadipocyte proliferation was affected in TNMD transgenic mice. Recently, Jeffery *et al*.^41^ demonstrated that there is a significant increase in preadipocyte proliferation during first week of HFD in visceral adipose tissue. To assess preadipocyte proliferation *in vivo*, we treated control and *Tnmd* transgenic mice with BrdU for 1 week in their drinking water and concurrently fed them HFD for 6 days (Fig. 4c). Preadipocytes were then isolated, seeded on coverslips and media selected for 24 h followed by immunostaining for BrdU and Pref1 as a preadipocyte marker. Remarkably, a significant increase in BrdU incorporation was observed in preadipocytes that were isolated from the eWAT of *Tnmd* transgenic mice (Fig. 4d). These data suggest that TNMD promotes eWAT expansion by enhancing preadipocyte proliferation in response to HFD. Consistent with these results, mRNA levels encoding adipogenic and lipogenic genes *Plin1*, sterol regulatory element-binding protein 1c (*Srebp1c*), fatty acid synthase (*Fasn*) and ATP-citrate lyase (*Acly*) were significantly increased, and the protein levels of PPARγ, PLIN, FASN and ACLY were 1.5–1.9-fold increased in *Tnmd* transgenic mouse visceral adipose tissue (Fig. 4e,f). These observations suggest that overexpressing *Tnmd* in visceral adipose tissue increases its storage capacity by both increasing adipocyte number and upregulating lipogenesis. Although no difference in [^14^C]-glucose incorporation into triglyceride was observed in adipose tissue explants that had been isolated from chow-fed control and TNMD transgenic animals (Supplementary Fig. 6a), such assays performed *in vitro* are quite artificial and may not represent physiological conditions.

### Reduced eWAT inflammation and fibrosis in *Tnmd* mice

**Figure 5:**
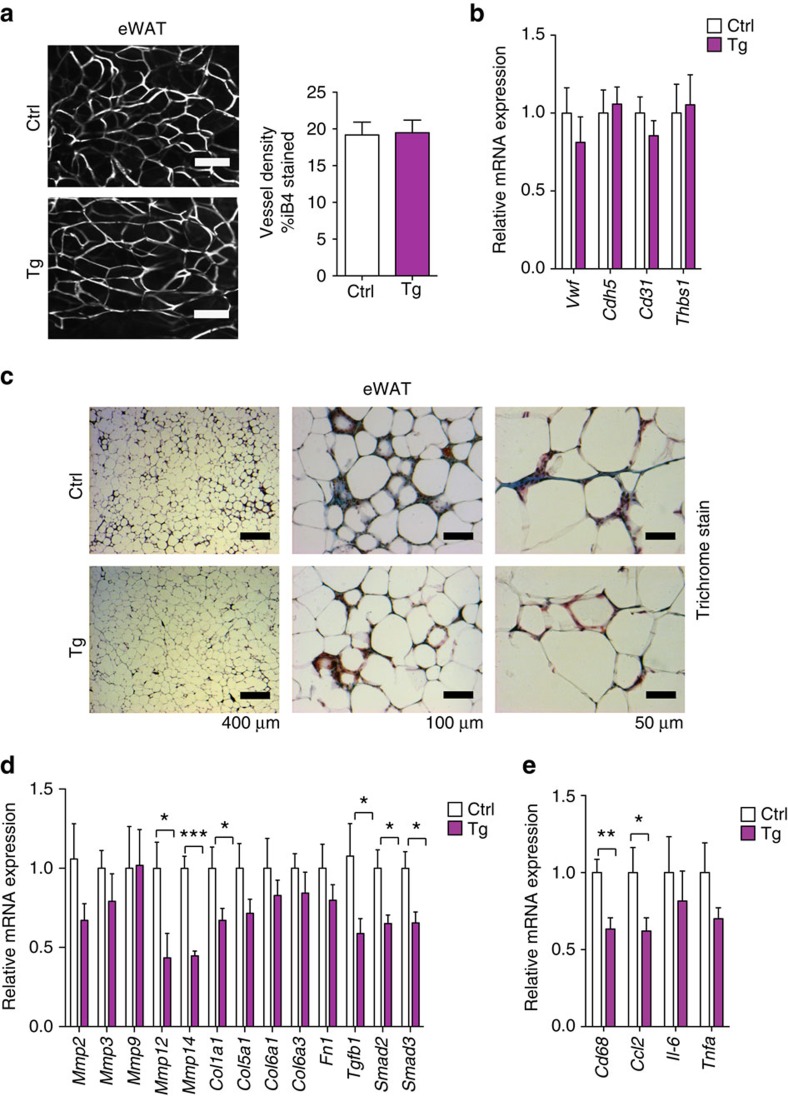
Decreased inflammation and collagen expression in eWAT of *Tnmd* transgenic mice. (**a**) Isolectin staining and vessel density quantification in eWAT from control and transgenic animals fed with 8 weeks of HFD (mean±s.e.m.; *n*=5 for both control and transgenics, *P<0.05, **P<0.01, by Student's *t*-test.) Scale bars, 100 μm. (**b**) Quantitative PCR for angiogenesis markers in eWAT from 16 weeks HFD-fed control and transgenic mice (mean±s.e.m.; *n*=9 (control), *n*=11 (transgenic), *P<0.05, **P<0.01, by Student's *t*-test). (**c**) Representative images of trichrome staining in eWAT from control (Ctrl) and transgenic (Tg) mice that had been fed HFD for 16 weeks. Scale bar, 400 μm (left panel); 100 μm (middle panel); 50 μm (right panel). (**d**) Quantitative PCR of ECM and transforming growth factor beta signalling genes in eWAT from HFD-fed animals (mean±s.e.m.; *n*=9 (control), *n*=10 (transgenic); *P<0.05, **P<0.01, by Student's *t*-test). (**e**) qRT–PCR analysis of inflammatory genes in eWAT from HFD-fed animals (mean±s.e.m.; *n*=9 (control), *n*=11 (transgenic); *P<0.05, **P<0.01, by Student's *t*-test).

A previous report suggested that TNMD has antiangiogenic properties in cultured endothelial cells^45^. However, *TNMD* knockout mice did not display any obvious vascular abnormalities^46^. These previous studies suggested that TNMD might have effects on blood vessel density or extracellular matrix (ECM) composition. Blood vessel morphology and density was assessed in HFD-fed control and TNMD transgenic mice; however, no differences were observed (Fig. 5a). Furthermore, endothelial cell marker gene expression was unaltered in TNMD transgenic adipose tissue as assessed by qRT–PCR (Fig. 5b).

Interestingly, a previous report characterizing TNMD null mice noted disorganized collagen fibrils^46^; thus, we hypothesized that TNMD might be involved in ECM processing. Trichrome staining was performed in control and TNMD transgenic mice to investigate whether TNMD is involved in regulating ECM and tissue fibrosis in eWAT. Remarkably, whereas abundant blue collagen staining was observed in eWAT of control mice, collagen accumulation was clearly decreased in transgenic mice, even in the inflamed areas (Fig. 5c). Moreover, gene expression analysis of whole adipose tissue revealed that genes encoding ECM proteins such as *Col1a1*, *Mmp12*, *Mmp14* as well as genes involved in transforming growth factor beta signalling were significantly decreased in transgenic eWAT compared with control animals suggesting that TNMD might be involved in regulating ECM composition (Fig. 5d). Therefore, TNMD may promote healthy visceral adipose tissue expansion through direct interaction with ECM proteins and regulation of ECM remodelling.

Many studies have demonstrated that inflammation, immune cell infiltration and expansion occur in visceral adipose tissue during obesity, which is associated with metabolic dysfunction and insulin resistance^2^,^21^,^24^,^25^,^47^,^48^,^49^. After prolonged HFD, immune cell infiltration was increased in eWAT of control mice, whereas both transgenic lines displayed fewer crown-like structures by histological analysis (Figs 4a and 5c; Supplementary Fig. 7). Furthermore, qRT–PCR results demonstrated that macrophage marker *Cd68* and macrophage-derived cytokines such as monocyte chemotactic protein 1 (*Ccl2*) were downregulated by 40% in transgenic mice (Fig. 5e). Thus, *Tnmd* overexpression also promotes adipose tissue integrity by preventing adipose tissue inflammation in obesity.

### TNMD inhibited lipid deposition in liver and BAT

**Figure 6:**
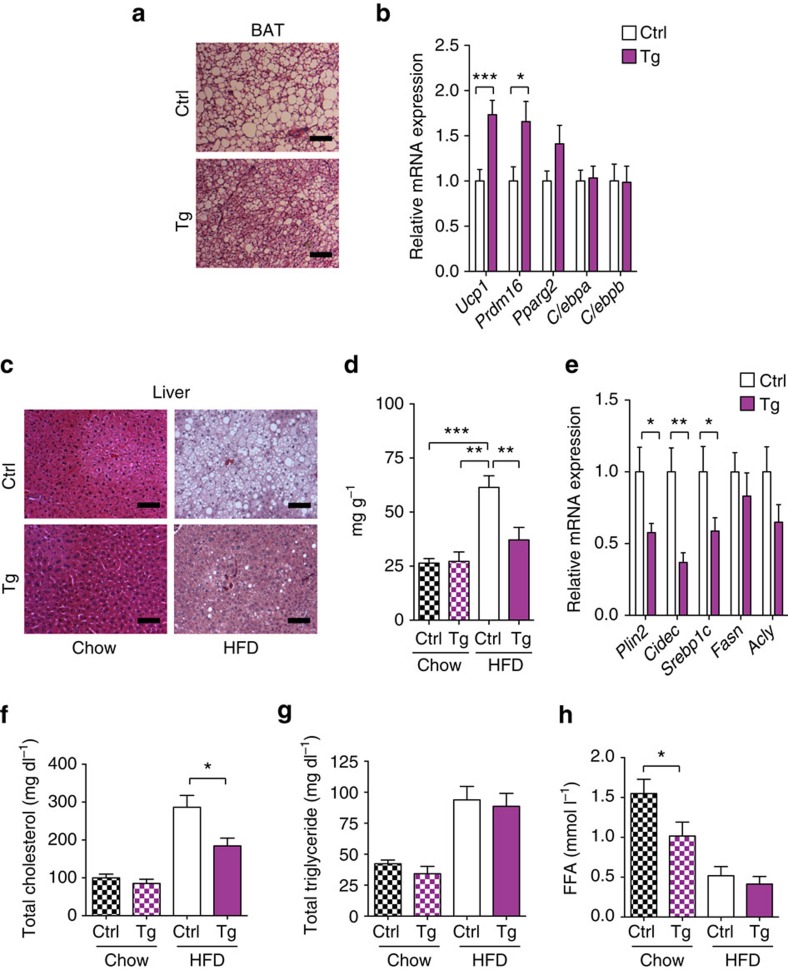
Peripheral lipid deposition was reduced in *Tnmd* transgenic mice. (**a**) Representative histological analysis of BAT from HFD-fed animals. Scale bar, 100 μm. (**b**) Quantitative PCR analysis of *Ucp1*, *Prdm16*, *Pparg2*, *C/ebpa* and *C/ebpb* in BAT of HFD-fed animals (mean±s.e.m.; *n*=9 (control), *n*=11 (transgenic); *P<0.05, **P<0.01, ***P<0.001, by Student's *t*-test). (**c**,**d**) Livers were isolated from animals fed chow or HFD for 16 weeks. (**c**) Haematoxylin and eosin staining of liver tissue. Scale bar, 100 μm. (**d**) Triglyceride content was measured (mean±s.e.m.; *n*=6 (control), *n*=8 (transgenic); *P<0.05, **P<0.01, ***P<0.001, by Student's *t*-test). (**e**) Gene expression in livers of HFD-fed animals (mean±s.e.m.; *n*=9 (control), *n*=11 (transgenic); *P<0.05, **P<0.01, ***P<0.001, by Student's *t*-test). (**f**) Total cholesterol (mean±s.e.m.; chow: *n*=12 (control), *n*=9 (transgenic); HFD: *n*=10 for both group; *P<0.05, **P<0.01, ***P<0.001, by Student's *t*-test). (**g**) Total triglyceride (mean±s.e.m.; chow: *n*=12 (control), *n*=10 (transgenic); HFD: *n*=7 for both group; *P<0.05, **P<0.01, ***P<0.001, by Student's *t*-test). (**h**) Free fatty acid levels (mean±s.e.m.; chow: *n*=9 (control), *n*=7 (transgenic); HFD: *n*=10 (control), *n*=11 (transgenic); *P<0.05, **P<0.01, ***P<0.001, by Student's *t*-test) were assessed in plasma samples from control (Ctrl) and transgenic (Tg) animals after 16 weeks of chow (dashed columns) or HFD (solid columns).

Consistent with our observation that the *Tnmd* transgenic mouse BAT depot was smaller, we observed fewer lipid droplets in histological samples of BAT (Fig. 6a). Importantly, brown adipocyte markers such as uncoupling protein 1 (*Ucp1*) and PR domain containing 16 (*Prdm16*) were significantly increased by nearly twofold in transgenic mouse BAT compared with that from controls upon HFD challenge (Fig. 6b). These results suggested that *Tnmd* overexpression in BAT may also promote BAT maintenance of mitochondrial fatty acid oxidation during HFD and contribute to overall beneficial metabolism. However, metabolic cage analysis in HFD-fed TNMD transgenic and control mice revealed no significant differences in respiratory exchange ratio (RER) or VO_2_ consumption (Supplementary Fig. 8c,d).

A significant decrease in liver weight was also observed in *Tnmd* transgenic mice compared with their control littermates (Fig. 3e). Therefore, we assessed hepatic lipid content in HFD-fed mice by both histological and triglyceride analysis. Although hepatic triglyceride content increased by twofold in control animals fed HFD, strikingly, *Tnmd* transgenic animals displayed no HFD-induced increase in hepatic triglyceride content and instead displayed a 60% reduction compared with control HFD-fed littermates (Fig. 6c,d). Consistent with the decreased lipid content of livers in the HFD-challenged transgenic animals, hepatic genes involved in lipid droplet formation that are associated with fatty liver such as *Plin2* and cell death-inducing DNA fragmentation factor alpha (DFFA)-like effector c (*Cidec*) were significantly downregulated in HFD-fed *Tnmd* transgenic mice (Fig. 6e).

Assessment of serum metabolic parameters demonstrated that HFD-fed *Tnmd* transgenic animals had significantly less total plasma cholesterol levels. However, no differences were detected in triglyceride levels on chow or HFD (Fig. 6f,g). Furthermore, serum-free fatty acid levels were significantly reduced in chow-fed *Tnmd* transgenic animals (Fig. 6h). However, though serum fatty acid levels can be a reflection of adipose tissue lipolysis, *ex vivo* lipolysis was not affected basally or after isoproterenol stimulation in *Tnmd* transgenic mice compared with littermate controls (Supplementary Fig. 9a). These data suggest that adipose tissue *Tnmd* overexpression may have a paracrine effect to regulate serum lipid concentrations.

### Improved insulin signalling in *Tnmd* overexpressing mice

**Figure 7:**
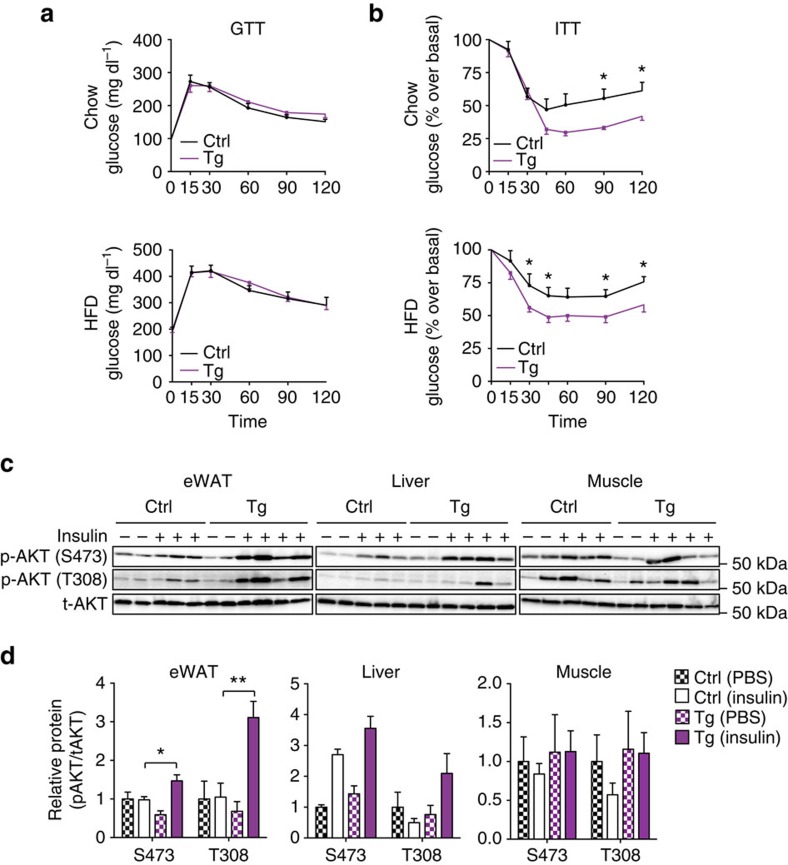
TNMD increased Akt phosphorylation in eWAT and improved systemic insulin sensitivity. (**a**) Glucose tolerance and (**b**) insulin tolerance tests were performed in male control (Ctrl) or transgenic (Tg) mice after 12 weeks of chow or HFD (mean±s.e.m.; chow: *n*=12 (control), *n*=10 (transgenic); HFD, *n*=11 (control), *n*=13 (transgenic); *P<0.05, **P<0.01, ***P<0.001, by Student's *t*-test). (**c**,**d**) Male control (Ctrl) and transgenic (Tg) mice that were fed with HFD for 12 weeks. Mice were fasted for 4 h and tissues were collected 15 min after PBS or insulin injection. (**c**) Western blot analysis and relative protein levels for p-Akt (S473), p-Akt (T308) and total Akt in eWAT, liver and muscle tissue lysates. (**d**) Densitometric analysis of **c** (mean±s.e.m.; *n*=4 (control and transgenic, PBS), *n*=5 (control, insulin), *n*=7 (transgenic, insulin), *P<0.05, **P<0.01, ***P<0.001, by Student's *t*-test).

Because *Tnmd* transgenic mice displayed decreased adipose tissue inflammation and less liver triglyceride content, we hypothesized that these animals might demonstrate improved insulin sensitivity. Although *Tnmd* transgenic mice displayed unaltered glucose tolerance in an intraperitoneal glucose tolerance test on chow or HFD (Fig. 7a), they were remarkably more insulin responsive than their control littermates on both chow and HFD during an insulin tolerance test (Fig. 7b). These data suggest that adipose-specific overexpression of TNMD improves systemic insulin sensitivity.

**Figure 8:**
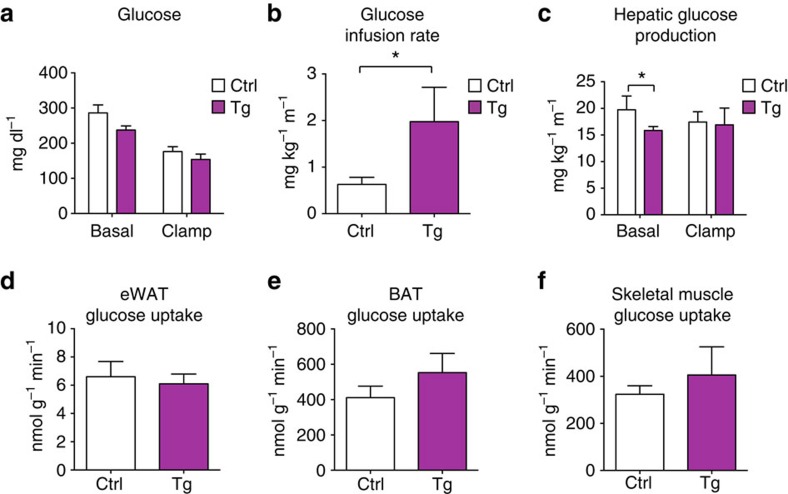
TNMD improved glucose homeostasis in mice with diet-induced obesity. Male control (Ctrl) and transgenic (Tg) mice were fed HFD for 12 weeks. Mice were fasted for 4 h before the clamp study. (**a**) Glucose levels before and during the hyperinsulinemic–euglycemic clamp (mean±s.e.m.; *n*=8 (control), *n*=4 (transgenic)). (**b**) Glucose infusion rate. (**c**) Hepatic glucose production measured during clamp (mean±s.e.m.; *n*=8 (control), *n*=4 (transgenic); *P<0.05, **P<0.01, ***P<0.001, by Student's *t*-test). Glucose uptake by (**d**) epididymal adipose tissue, (**e**) brown adipose tissue (interscapular) and (**f**) skeletal muscle (gastrocnemius) (mean±s.e.m.; *n*=8 (control), *n*=4 (transgenic)).

To determine whether insulin sensitivity was also enhanced in *Tnmd* transgenic mice at the molecular level in insulin-responsive tissues, HFD-fed control and *Tnmd* transgenic mice were injected with either insulin or PBS. Then, 15 min later, muscle, liver and eWAT were isolated from animals and analysed for phospho-Akt levels as an indicator of insulin signalling. Akt phosphorylation at both S473 and T308 sites as detected by specific anti-phosphoserine and anti-phosphothreonine antibodies was significantly increased in *Tnmd* transgenic mouse eWAT compared with littermate controls. Moreover, a trend towards an increased Akt phosphorylation at these sites was observed in both liver and muscle, suggesting that in addition to improving adipose tissue insulin sensitivity, adipose TMND might also improve insulin responsiveness in other peripheral tissues (Fig. 7c,d). To further assess what tissues contributed to the enhanced insulin sensitivity in *Tnmd* transgenic mice, hyperinsulinemic–euglycemic clamps were performed. The clamp data demonstrated that although glucose levels during the clamp were similar, *Tnmd* transgenic animals had higher insulin sensitivity compared with control littermates as assessed by glucose infusion rate (Fig. 8a,b). This difference could be attributed to improved hepatic insulin sensitivity because *Tnmd* transgenic animals displayed decreased hepatic glucose production (Fig. 8c), whereas no differences were observed in tissue-specific glucose uptake (Fig. 8d,f). Collectively, these data suggest that *Tnmd* overexpression in murine adipose tissue improves systemic insulin sensitivity.

## Discussion

Identification of factors that modulate pathological consequences of obesity is a vital step towards development of novel therapeutic approaches to treatment of insulin resistance and other aspects of metabolic syndrome. In this study, we demonstrated that insulin-resistant obese individuals have increased *TNMD* expression compared with insulin-sensitive controls in the omental adipose depot, even when matched for BMI (Fig. 1b,c). Previous studies demonstrated that *TNMD* is highly expressed in human adipose tissue and its expression is further increased in obese conditions^31^. Furthermore, genetic studies that investigated an association between single-nucleotide polymorphisms and various metabolic markers suggested a potential role for this gene in adipose tissue in disease^37^. Though no difference in *TNMD* expression was observed in insulin-resistant versus insulin-sensitive patients in subcutaneous adipose tissue depots, previous studies that analysed subcutaneous adipose tissue biopsies demonstrated a correlation of *TNMD* expression, fasting serum insulin levels and homeostatic model assessment-insulin resistance (HOMA-IR) in obese patients^31^,^33^. *TNMD* expression is predominantly in adipocytes compared with the SVF (Fig. 1d), suggesting that the expression differences observed in insulin-sensitive versus insulin-resistant individuals mainly result from expression changes in primary adipocytes.

A key finding in this study is that TNMD is required for differentiation of human SGBS and mouse 3T3-L1 preadipocytes (Fig. 2). Because *TNMD* expression is readily stimulated after adipogenic induction and continues to increase during differentiation in human cells, we supposed that the absence of *TNMD* in preadipocytes would impair early differentiation. Consistent with this notion, when *TNMD* expression was silenced, expression of transcription factors involved in adipogenesis such as *C/EBPA* and *PPARG* was decreased at early time points of differentiation (Fig. 2f,g), and adipogenesis was impaired. While the exact function of TNMD in this process is unclear, TNMD is hereby identified as a novel required factor in early stages of adipocyte differentiation. *TNMD* expression is actually decreased 2 days after induction of 3T3-L1 cell adipogenesis. Therefore, unlike the case in human cells, its expression is actually higher in mouse preadipocytes when compared with mature adipocytes. However, silencing of TNMD before induction was sufficient to inhibit the adipogenesis of these mouse preadipocytes, showing that TNMD is required for the initiation of adipogenesis in both species.

Because it appeared TNMD had a potential role in human cells and human patients but was expressed at low levels in mouse adipose tissue, we sought to address the role of TNMD in adipose tissue by generating a mouse model with higher adipose tissue-specific *Tnmd* expression. Such adipose-specific *Tnmd* expression in transgenic mice increased adipogenic and lipogenic gene and protein expression in eWAT upon HFD feeding. Notably, PPARγ, one of the major regulators of glucose metabolism and adipocyte function^50^,^51^,^52^, was significantly upregulated in the eWAT of HFD-fed *Tnmd* transgenic mice compared with their control littermates (Fig. 4f). Because activation of PPARγ has many beneficial effects on adipose tissue including improving lipid metabolism and decreasing serum free fatty acids (FFAs)^53^,^54^,^55^, it can be inferred that TNMD strongly influences adipogenesis through regulating PPARγ expression *in vivo.* Consistent with these findings *in vivo*, silencing *TNMD* significantly reduced adipocyte differentiation and adipogenic gene expression, including *PPARG* in human and mouse preadipocytes *in vitro*.

Many recent studies have described that the ECM has a critical function to regulate adipose tissue homeostasis in obesity^56^,^57^,^58^,^59^,^60^, and exogenous signals regulated by ECM proteins are involved in determining the fate of mesenchymal progenitor cells. For example, ECM stiffness and composition regulates Wnt and transforming growth factor beta signalling, which has an inhibitory or, in some cases, stimulatory role in adipogenesis^15^. Adipose tissue ECM also provides a suitable environment for changes in cell shape during adipogenesis and cell expansion^61^,^62^,^63^,^64^. In this study, we noted a reduction in collagen staining in *Tnmd* transgenic adipose tissue, and collagen and matrix metalloproteinase gene expression was also significantly reduced in *Tnmd* overexpressing adipose tissue (Fig. 5). These results suggest that TNMD may promote beneficial adipogenesis at least in part by modulating properties of the ECM in adipose tissue.

In addition to enhancing insulin signalling in eWAT, beneficial effects of TMND in the liver was also observed, which enhanced systemic insulin sensitivity (Figs 6 and 7). *Tnmd* transgenic mice had reduced hepatic lipid deposition and were more responsive to insulin even in lean, chow-fed conditions. Furthermore, hepatic glucose production was reduced in TNMD transgenic animals (Fig. 8). However, it is not established whether these peripheral effects are due to the improved lipid sequestration and decreased inflammation in eWAT. Future studies will investigate the mechanisms by which adipose TNMD function might cause beneficial signalling to other tissues.

*Tnmd* transgenic mice had smaller BAT with fewer lipid droplets after HFD (Figs 3e and 6a). Furthermore, thermogenic genes *Ucp1*, *Prdm16* and *Pparg* were upregulated in transgenic animals compared with their littermate controls (Fig. 6b). PRDM16 and PPARγ are not only involved in BAT differentiation but also in BAT maintenance along with UCP1 (refs 65, 66). However, though TNMD was overexpressed in BAT in this model, thermogenic capacity of the animals as assessed by RER remained unchanged (Supplementary Fig. 8d). Future studies will utilize thermoneutral or cold challenge conditions to assess whether TNMD has a role in regulation of BAT energy expenditure.

*TNMD* expression was higher in the human insulin-resistant cohort in our study, yet paradoxically mice overexpressing *Tnmd* in adipose tissue displayed improved insulin sensitivity (Figs 7 and 8). In humans, omental fat from insulin-resistant subjects displays more inflammation and larger adipocytes compared with BMI-matched insulin-sensitive subjects^2^. It is possible that the increased inflammation in these insulin-resistant subjects arises from enhanced adipocyte death in the insulin-resistant omental fat. Indeed, a correlation between cell death, insulin resistance and adipocyte size has previously been reported^67^. Thus, we speculate that TNMD might be increased in insulin-resistant omental fat to increase adipocyte replenishment in these conditions^42^,^68^. Indeed, increased preadipocyte proliferation was observed in TNMD transgenic animals (Fig. 4), which could promote healthy tissue expansion. It is also possible that either the omental fat microenvironment or endocrine signals associated with the insulin-resistant state can contribute to *TNMD* overexpression, perhaps as a compensatory mechanism to promote adipogenesis and increase insulin responsiveness. Finally, most of the insulin-resistant human subjects in our cohort had been treated for different amounts of time with various medications including thiazolidinediones before bariatric surgery and tissue collection. Thus, we cannot rule out the possibility that *TNMD* expression was increased in insulin-resistant subjects as a result of these medications.

In summary, our study reveals that the gene *TNMD*, which is highly expressed in human adipose tissue, encodes a protective adipose tissue factor that promotes preadipocyte proliferation, adipogenesis, adipose tissue health and insulin responsiveness *in vivo*. The data presented herein support the hypothesis that TNMD contributes to beneficial visceral adipose tissue expansion that protects against metabolic dysfunction. Because adipose TNMD expression improves insulin sensitivity systemically, it may have potential as a therapeutic target to protect metabolic homeostasis in obesity.

## Methods

### Animals

All of the studies were approved by The University of Massachusetts Medical School Institutional Animal Care and Use Committee. Mice were housed in an animal facility with a 12 h light/dark cycle and had access to water, chow or HFD (12492i Harlan) *ad libitum* during the indicated periods. For *in vivo* preadipocyte proliferation studies, mice were treated with 0.8 mg ml^−1^ BrdU in water with 1% sucrose. Water was changed every 72 h and kept in the dark. Mice were killed by CO_2_ and bilateral pneumothorax.

### Human samples

Human adipose tissue samples were collected from morbidly obese patients who underwent gastric bypass surgery between 2005 and 2009 at the University of Massachusetts Medical School were selected for this study^2^. Samples used for microarray analysis were from BMI-matched female patients, whereas qRT–PCR and western blot validations were performed in samples from both males and females. Adipose tissue samples were obtained from lower abdominal wall (for subcutaneous) and omentum (for visceral) during the surgery. Informed consent was given by the patients and the study was approved by University of Massachusetts Medical School Institutional Review Board.

### Generation of adiponectin-*Tnmd*-flag transgenic mice

Full-length mouse *Tnmd* with a C-terminal Flag tag was inserted 3′ to 5.4-kb adiponectin promoter at the Cla I site^69^. After verifying both ends by sequencing, the transgenic cassette was linearized by Kpn1 and Xho1 digestion, purified and submitted for pro-nuclear injection. The transgene was introduced into embryos from C57BL/6J mice (000058; Jackson Laboratories). Embryos were then implanted into pseudopregnant C57BL6/J females by the UMASS Transgenic Animal Facility. Male transgenic animals were crossed with C57BL6/J females. Genotyping was performed by PCR from genomic DNA with the following primers: 5′- GACCAGAATGAGCAATGGGTG , 3′- ATCGTCGTCATCCTTGTAGTCG -3′. Six- to eight-week-old Adiponectin-*Tnmd*-Flag transgenic mice and age-matched wild-type littermates were used in the experiments. Male animals were used unless otherwise was stated.

### Western blotting

Cell lysates were prepared using RIPA buffer (150 mM NaCl, 50 mM Tris pH 7.4, 1% sodium deoxycholate, 1% NP-40, 0.2% SDS, 50 mM EDTA) containing 1X HALT protease and phosphatase inhibitors (Thermo Scientific). Total protein was separated on SDS–polyacrylamide gel electrophoresis gels and transferred to nitrocellulose membranes. Membranes were blocked with 5% milk solution in TBS-T and immunoblotted with an antibody generated against NGIEFDPMLDERGYC peptide from C terminus of TNMD (Rockland, 1:5,000) and antibodies against C/EBPα (8178), C/EBPβ (3082), PPARγ (2443), phospho-AKT(S308) (9275), phospho-AKT(T473) (4060), total AKT (4691 or 2920), ACLY (4332), ACC (3662), PLIN (3470), (Cell Signaling, 1:1,000), FASN (BD Biosciences, 610963, 1:2,000), FLAG (F7425, 1:2,000) tubulin and actin (Sigma, 1:5,000). Uncropped gel pictures are provided in Supplementary Figs 10 and 11.

### qRT–PCR analysis

Total RNA was isolated using TriPure (Roche) according to the manufacturer's protocol. DNAse (DNA-free, Life Technologies)-treated RNA was reverse transcribed into complementary DNA using iScript (BioRad). Quantitative PCR analyses were performed using SYBR green (iQ SYBR Green Supermix, BioRad) on BioRad CFX97. Primer sequences used for qRT–PCR analyses were listed in Supplementary Table 2. HUGO Gene Nomenclature Committee's Guideline was used for gene names written in this manuscript.

### Cell culture

SGBS cells were obtained from Dr Martin Wabitsch's laboratory and cultured in DMEM/F12 media supplemented with 10% fetal bovine serum (FBS), 33 μM biotin, 17 μM pantothenic acid 100 U ml^−1^ penicillin and 0.1 mg ml^−1^ streptomycin until full confluence. Cells were washed with PBS before differentiation was stimulated with serum-free media containing rosiglitazone, dexamethasone, 3-isobutyl-1-methylxanthine, cortisol, transferrin, triiodotyronin and human insulin. Four days later, the differentiation cocktail was replaced with adipocyte maintenance media (DMEM/F12, biotin, pantothenic acid, transferrin, insulin and cortisol). Cells were maintained until they are fully differentiated (day 14). 3T3-L1 preadipocytes were obtained from AATC (CL-173). Cells were cultured in high-glucose DMEM media supplemented with 10% FBS, 50 μg ml^−1^ streptomycin and 50 U ml^−1^ penicillin and differentiated into adipocytes with high-glucose DMEM media with 10% FBS, 50 μg ml^−1^ streptomycin, 50 U ml^−1^ penicillin, 5 μg ml^−1^ insulin, 0.25 μM dexamethasone and 0.5 mM 3-isobutyl-1-methylxanthine.

### Oil Red O staining

Differentiated adipocytes were fixed with 10% formalin, washed with 60% isopropanol and air dried. Oil Red O working solution was added on cells and washed with distilled water to remove excess dye. Oil Red O was extracted using 100% isopropanol and absorbance was measured in 520 nm in spectrometer for quantification.

### siRNA transfection of SGBS and 3T3-L1 preadipocytes

SGBS or 3T3-L1 preadipocytes were plated into 12-well plates (10^5^ cells per well) and transfected with 100 nM (SGBS) or 50 nM (3T3-L1) scrambled siRNA or TNMD siRNA (Dharmacon, siGENOME, Smartpool) using Lipofectamine RNAiMax (Life Technologies) according to the manufacturer's protocol. Forty-eight hours after transfection, cells were collected or stimulated for adipogenesis. siRNA sequences for mouse and human *TNMD* were provided in Supplementary Table 3.

### MTT assay

SGBS preadipocytes (10^4^ cells per well) were plated in 96-well plates. Twenty-four hours later, they were transfected with either scrambled or TNMD siRNA. Cell viability was measured 0, 24 and 48 h after transfection using an MTT assay kit (Biotium Inc.) according to the manufacturer's instructions.

### Immunofluorescence staining

Differentiated adipocytes were fixed with 10% formalin and permeabilized in 0.1% Triton X in PBS. Bovine serum albumin (BSA; 3%) in PBS was used for blocking. Cells were incubated overnight with PLIN antibody (Cell Signaling) diluted in 1% BSA solution and 45 min at 37 °C with secondary antibody (Alexa Fluor 488, Life Technologies). Nuclei were stained with 4,6-diamidino-2-phenylindole. Preadipocytes were fixed in 70% cold methanol and treated with 1.5 M HCl for 30 min. After washing, they were washed and blocked with 5% normal goat serum and 0.3% Triton X-100 in PBS. Primary antibodies for BrdU and Pref1 (Cell Signaling, 5292, 1:1,000 and EMD Millipore, AB3511, 1:100) were applied overnight. Cells were washed and incubated with secondary antibodies for 90 min at room temperature and mounted with Prolong Gold Antifade Reagent with 4,6-diamidino-2-phenylindole. Images from at least three different areas were taken, and nuclei were counted by Image J Analysis Software. For vessel density in adipose tissue, whole-mount staining was performed after 10% formalin fixation. Tissues were blocked overnight in 10% BSA and 0.3% triton X-100 in PBS at 4 °C, stained overnight with Isolectin B4 (Life Technologies I21411; 1:40) in 100 mM MgCl_2_, 100 mM CaCl_2_, 10 mM MnCl_2_ and 1%Triton X-100 in PBS at 4 °C, and washed three times at 20 min in 5% BSA, 0.15% triton X-100 in PBS at room temperature. Approximately, 1-mm cubes were whole mounted in ProLong Gold (Life Technologies). Images were visualized in flattened 25-μm z-stacks with confocal microscopy at × 10. Images were acquired with MetaMorph Software, version 6.1 (Universal Imaging, Downingtown, PA). At least three technical replicates of adipose tissue images were quantified per mouse and averaged for average vascular density. Images were quantified using Image J Analysis Software.

### Histology

Tissue samples were fixed in 10% formalin and embedded in paraffin. Sectioned slides then stained with haematoxylin and eosin by the UMass Medical School Morphology Core. Adipocyte size was assessed using Adiposoft software^70^.

### Insulin and glucose tolerance tests

Mice fed with the indicated diets were fasted 16 hours for glucose tolerance tests and 4 hours for insulin tolerance tests. Basal blood glucose was measured with a Breeze-2-glucose meter (Bayer) before and after they were intraperitoneally injected with glucose (1 g kg^−1^) or insulin (1 IU kg^−1^).

### Hyperinsulinemic–euglycemic clamp and metabolic cage studies

The clamp and metabolic cage studies were performed at the UMass Mouse Metabolic Phenotyping Center. Mice fed with HFD for 12 weeks were subjected to a 4-h fast, and a 2-h hyperinsulinemic–euglycemic clamp was performed with a primed and continuous infusion of human insulin (150 mU kg^−1^ body weight priming followed by 2.5 mU kg^−1^ min^−1^; Humulin, Eli Lilly). During the clamp, 20% glucose was infused at variable rates to maintain euglycemia^71^. Whole-body glucose turnover was assessed with a continuous infusion of [3-^3^H]glucose, and 2-deoxy-D-[1-^14^C]glucose (PerkinElmer, Waltham, MA) was administered (10 μCi) at 75 min after the start of clamps to measure insulin-stimulated glucose uptake in individual organs. At the end of the study, mice were anaesthetized, and tissues were taken for biochemical analysis. The metabolic cages were used to measure food intake, RER, VO_2_ consumption and physical activity over a 3-day period, and average for each parameter was calculated (TSE Systems).

### Plasma analysis

Blood samples were collected from animals after a 16-h fast via cardiac puncture. Serum triglyceride levels were measured using a Serum Triglyceride Determination Kit (Sigma). Free fatty acid and total cholesterol in plasma samples were analysed using NEFA (Free Fatty Acid) Kit (Wako Diagnostics) and Cholesterol/Cholesteryl Ester Quantitation Kit (Abcam), respectively.

### *Ex vivo* lipogenesis assay

Adipose tissue explants were incubated with labelling media containing 2.50% fatty acid-free BSA, 1% (v/v) Pen/Strep, 0.5 mM D-glucose, 2 mM sodium pyruvate, 2 mM glutamine and 2 μCi ml^−1^ [^14^C]-U-glucose. Insulin (1 μM) was added to insulin-stimulated conditions and incubated for 4.5 h before lipid extraction at 37 °C. The reaction was stopped by adding modified Dole's extraction mixture (80 ml isopropanol, 20 ml hexane and 2 ml 1 N H_2_SO_4_). Total triglyceride was extracted with hexane, washed and evaporated, and counted by liquid scintillation.

### *Ex vivo* lipolysis assay

Adipose tissue explants were isolated from iWAT and eWAT of mice that had been fed chow diet for 12 weeks. Fat pads were measured, and a 30-mg piece was placed in freshly prepared KRH buffer (125 mM NaCl, 5 mM KCl, 1.8 mM CaCl_2_, 2.6 mM MgSO_4_ and 5 mM HEPES, pH 7.2) containing 2.5% BSA (fatty acid free) and 1 mM sodium pyruvate until stimulation. Subsequently, extracts were treated with PBS or isoproterenol (10 μM) for 2 h at 37 °C. Free glycerol content in the buffer was quantified for each sample using the Free Glycerol Determination Kit (Sigma). Glycerol release from each sample was normalized to the weight of each fat pad.

### Statistical analysis

A two-tailed Student's *t*-test with Welch's correction was performed to analyse the difference between two groups using Microsoft Excel or Graph Pad Prism 6.0. The Grubb's test was used to determine the statistical outliers. In case of an outlier was determined, it was removed from the statistical analysis. Experimental data were represented as the mean of at least three biological replicates. *P* values<0.05 were considered to be statistically significant. Variance was estimated using the s.e. of the mean for both group that are statistically compared. No statistical methods were used to predict sample size. No randomization or blinding was performed to allocate the samples for animal experiments.

## Additional information

**Accession codes:** Microarray data have been deposited in GEO database under accession code GSE76319 and GSE20950.

**How to cite this article:** Senol-Cosar, O. *et al*. Tenomodulin promotes human adipocyte differentiation and beneficial visceral adipose tissue expansion. *Nat. Commun.* 7:10686 doi: 10.1038/ncomms10686 (2016).

## Supplementary Material

Supplementary Information Supplementary Figures 1-11 and Supplementary Tables 1-3.
