# Supplementary material for: Tenomodulin promotes human adipocyte differentiation and beneficial visceral adipose tissue expansion
Source: Nat Commun. 2016 Feb 16;7:10686. doi: 10.1038/ncomms10686 (PMC4757769; doi:10.1038/ncomms10686)
Supplement: Supplementary Information — Supplementary Figures 1-11 and Supplementary Tables 1-3. [file ncomms10686-s1.pdf]

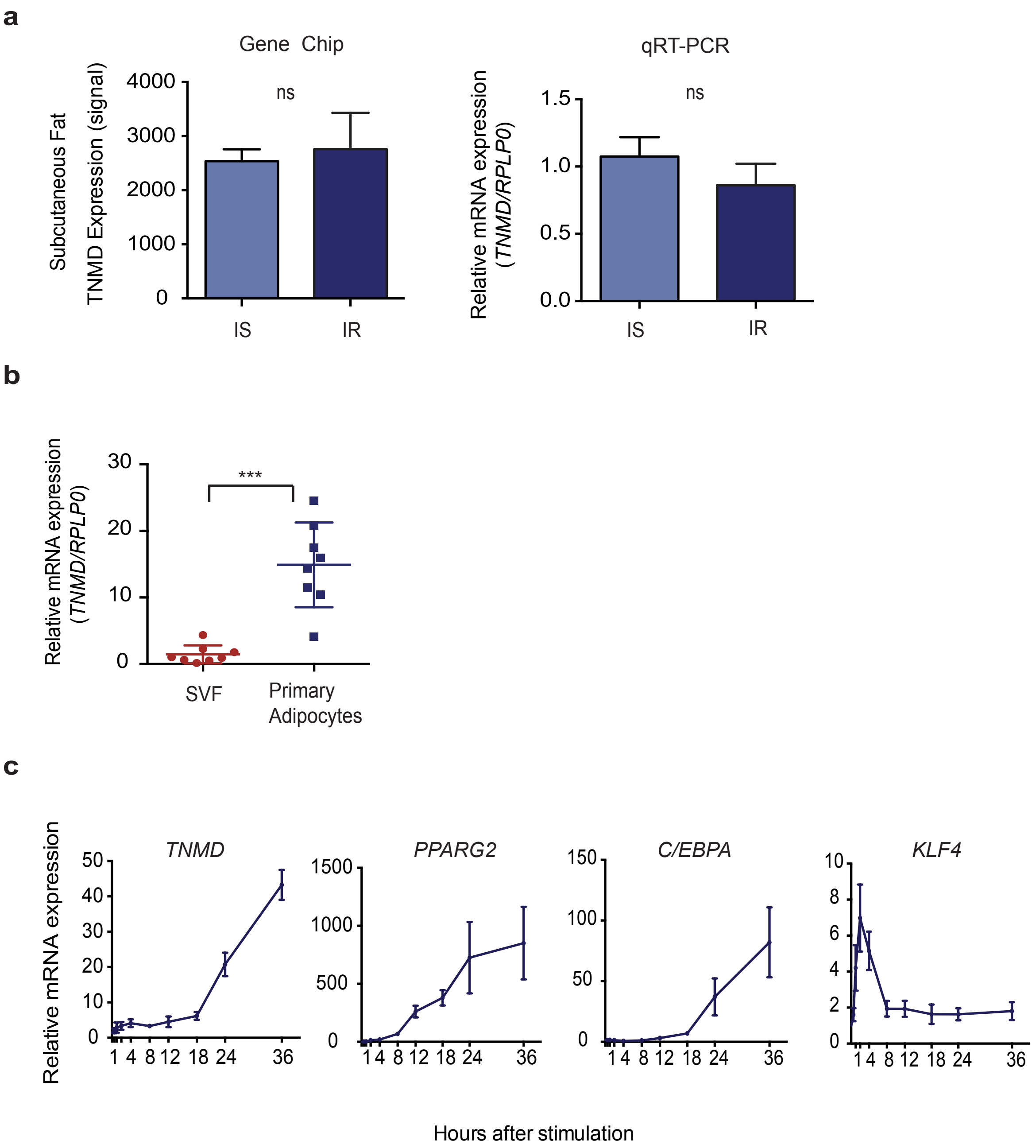

Supplementary Figure 1. *TNMD* expression in human subcutaneous adipose tissue and early time points of differentiation in human adipocytes

(a) *TNMD* expression was analyzed by microarray and quantitative PCR in subcutaneous adipose tissue depots (mean±SEM; n=7, insulin sensitive, n=8, insulin resistant, Student's t-test). **(b)** *TNMD* expression in the stromal vascular fraction or primary adipocytes isolated from human subcutaneous fat depot (mean±SEM; n=8 for both SVF and primary adipocytes, *p<0.05, **p<0.01, ***p<0.001, Student's t-test). **(c)** SGBS cells were induced to differentiate, RNA was isolated, and qRT-PCR was performed for *TNMD, PPARG2, C/EBPA* and *KLF4* expression at the indicated early time points (mean±SEM; n=3, Student's t-test).

a

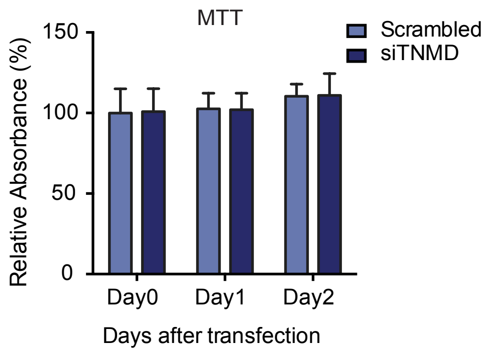

b

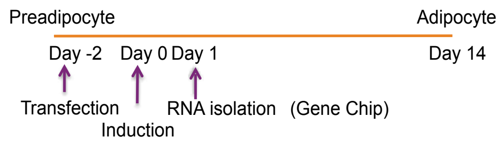

Fold change (d0 vs d1)

Gene	Scrambled	siTNMD	
PDK4	82.10	60.70	**
OLAH	39.26	21.29	*
ADH1B	37.16	25.78	*
IL18R1	25.96	13.43	
CIDEC	9.99	6.80	
ACACB	9.94	6.75	*
ZBTB16	9.35	4.10	
PLIN1	9.25	5.65	
LMO3	8.93	6.85	**
KLF15	7.80	4.35	
BMP6	6.78	5.12	*
ACSL1	6.14	4.67	
ZNF117	5.26	3.69	
PITPNC1	4.88	4.89	
PDE4D	4.79	4.73	
PIK3R1	4.78	3.56	*
PLIN4	4.38	2.89	
PPARG	4.17	2.91	**
TBX15	3.58	2.80	**
JAK2	3.22	1.69	
PC	3.21	2.47	**
FITM2	3.03	2.52	
STAT5A	1.49	1.34	*

c

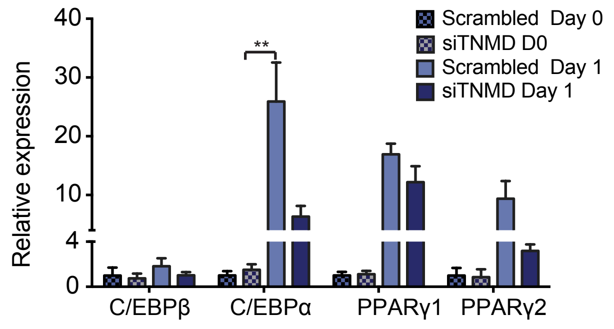

d

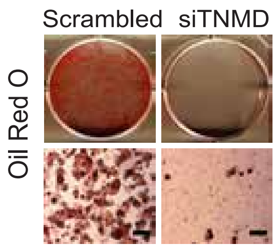

e

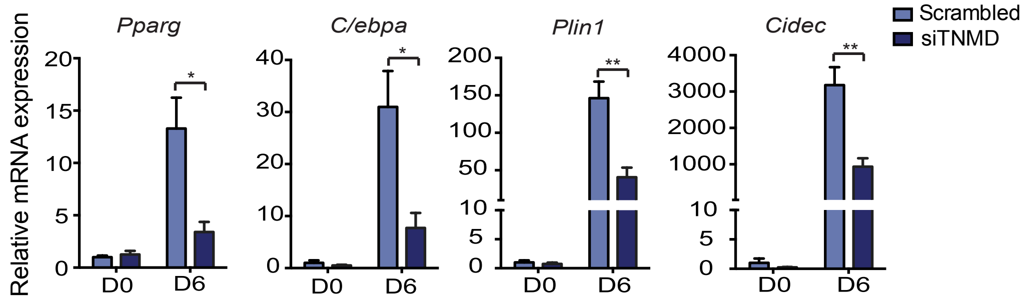

Supplementary Figure 2. Knockdown of TNMD attenuates differentiation

(a) MTT cell viability assay (mean± SEM; n=3) (b) Experimental design of microarray analysis. Fold change in various genes upon adipogenic stimuli and comparison among cells transfected with scrambled siRNA and siTNMD (n=3, *p<0.05, **p<0.01, ***p<0.001, Student's t-test). (c) Quantification of Western blot analysis from Figure 1g (n=3-6, *p<0.05, **p<0.01, ***p<0.001, Student's t-test). (d,e) 3T3-L1 adipocytes were treated with scrambled or TNMD siRNA and differentiated for 6 days (d) Representative Oil-red O staining in 3T3-L1 cells on day 6 of differentiation. Whole well pictures (upper panel) and light microscopy image. Scale bar represents 100μm (e) Quantitative PCR analysis for Pparg, C/ebpa, Plin1 and Cidec on day 0 and day 6 of differentiation (n=3, *p<0.05, **p<0.01, ***p<0.001, Student's t-test).

a

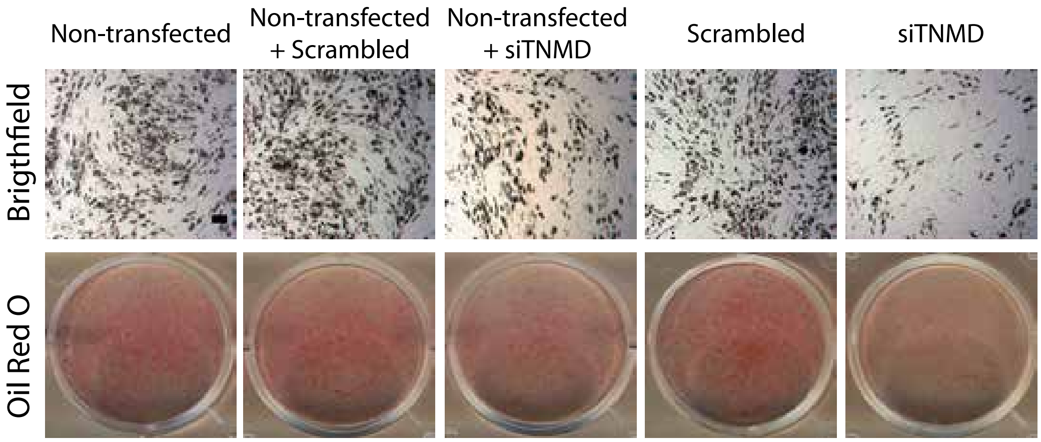

Supplementary Figure 3. Co-culturing non-transfected SGBS preadipocytes with TNMD transfected cells. (a) SGBS preadipocytes were transfected with either Scrambled or siTNMD. 24 hours after transfection, cells were trypsinized and seeded in equal numbers from stated groups in a different well. 2 days after transfection cells were stimulated with adipogenic coctail. Adipogenesis was analyzed by Oil Red O staining and microscopy. The data are representative of two independent experiments. Scale bar represents 100µm.

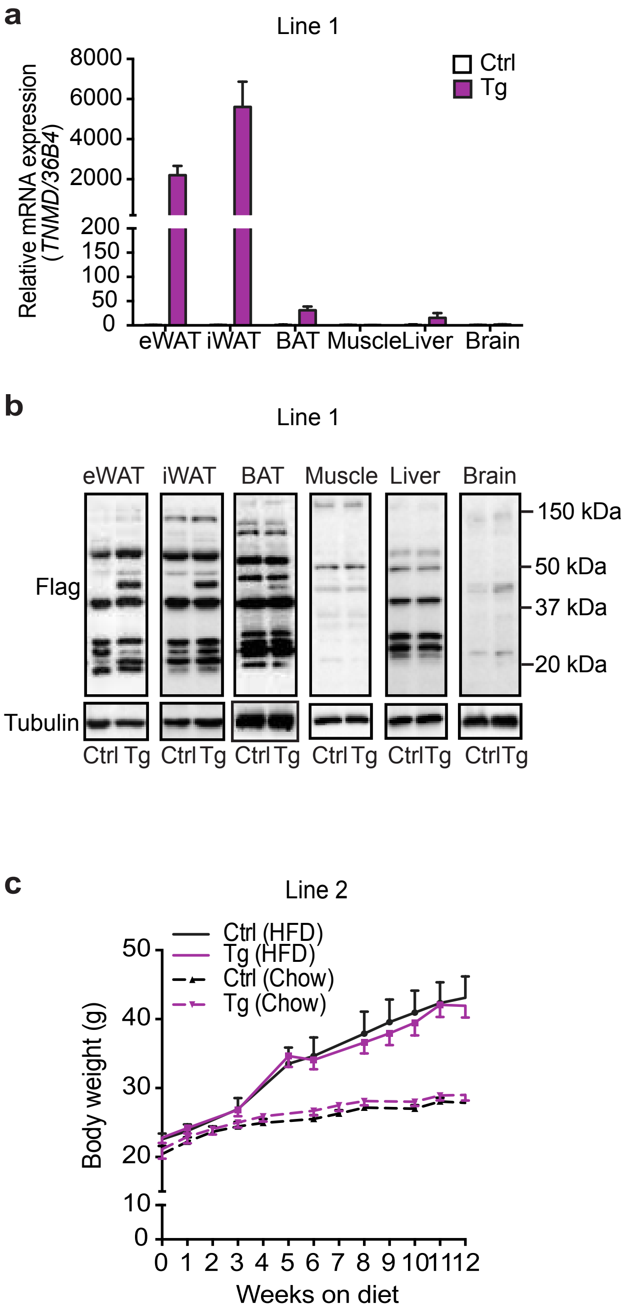

Supplementary Figure 4. *TNMD* expression in mouse tissues in Line 1 and Line 2
(a) Quantitative PCR analysis of *Tnmd* in eWAT, iWAT, BAT, Liver, Muscle and Brain isolated after 16 weeks of HFD in male control (Ctrl) and transgenic (Tg) mice (Line 1) (mean± SEM; n=5 (control), n=11 (transgenic), *p<0.05, **p<0.01, ***p<0.001, Student's t-test). **(b)** Whole gels for Western blot analysis of Flag and tubulin expression in eWAT, iWAT, BAT, liver, muscle and brain tissue lysates (Line 1, n=4 (control), n=9 (transgenic) in eWAT and iWAT n=4 (control) n=4 (transgenic) in BAT, Muscle, Liver and Brain). **(c)** Weight curves mice fed chow or HFD for 12 weeks (Line 2) (mean±SEM; Chow n=5 (control), n=7 (transgenic); HFD n=4 (control), n=7 (transgenic), Student's t-test).

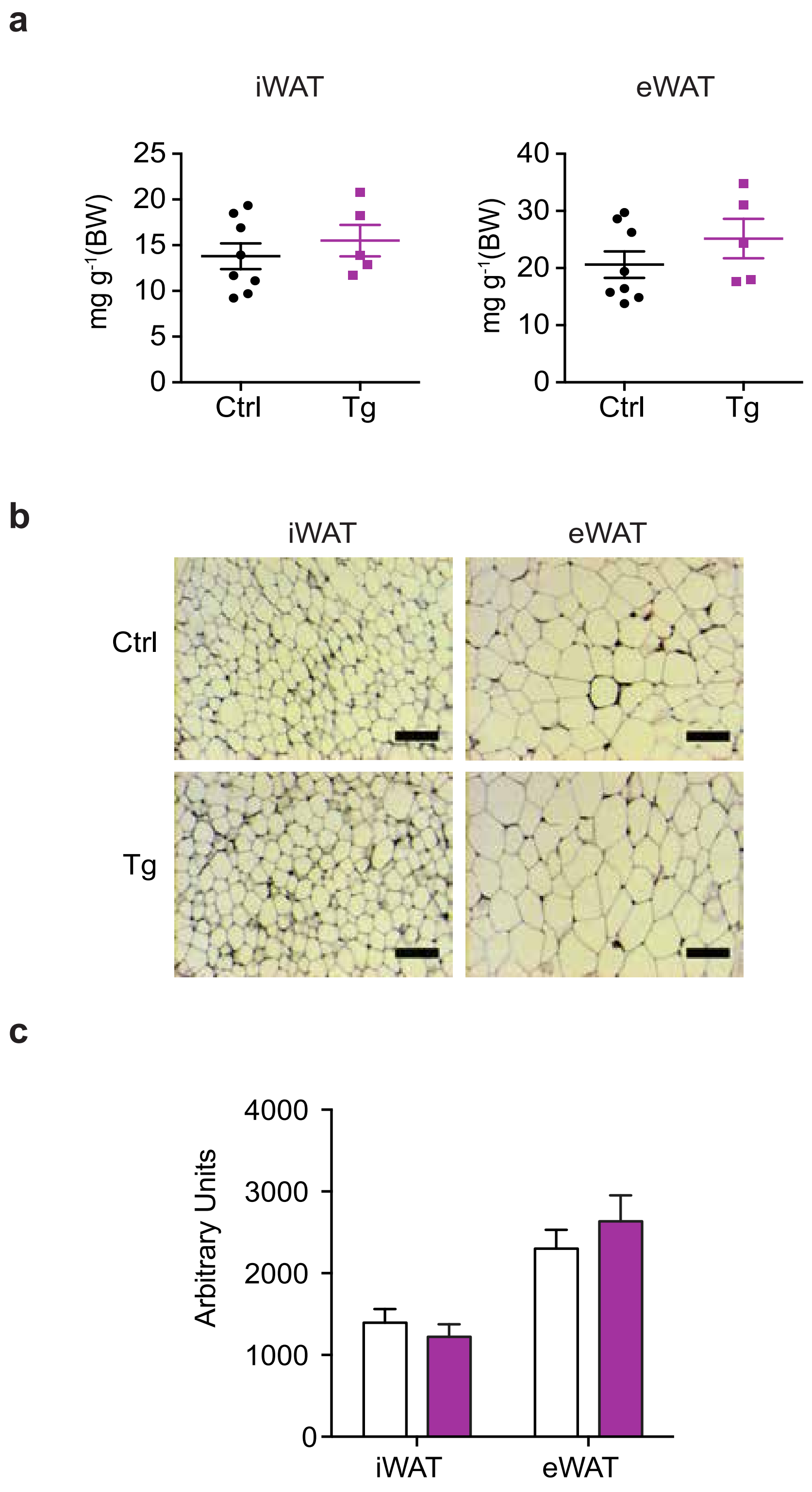

Supplementary Figure 5. Assesment of iWAT and eWAT depots at 4 weeks of HFD in control and transgenic mice. (a) Epididymal and inguinal white adipose tissue weights measured after 4 weeks of HFD in control (Ctrl) and transgenic (Tg) animals and normalized to total body weights (mean±SEM; n=8 (control), n=5 (transgenic), Student's t-test) **(b)** Histologic analysis of eWAT and liver of from transgenic (Tg) and control (Ctrl) mice after 4 weeks. Scale bars represent 100 μm **(c)** Adipocyte size was analyzed and avarage adipocyte size was calculated for each group (mean±SEM; n=8 (control), n=5 (transgenic), Student's t-test).

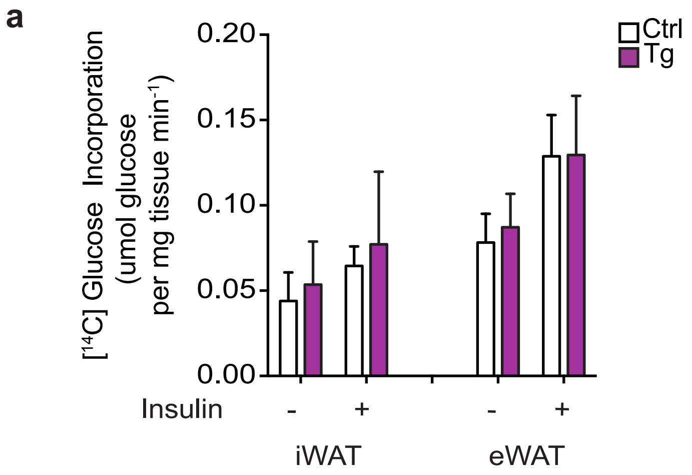

Supplementary Figure 6. Ex vivo lipogenesis analysis in adipose depots in transgenic and control mice. (a) Insulin stimulated [^{14}C]-Glucose incorporation into triglyceride was measured in adipose tissue explants that had been isolated from 12 weeks chow fed mice (mean±SEM; n=4 for both control and transgenic, Student's t-test).

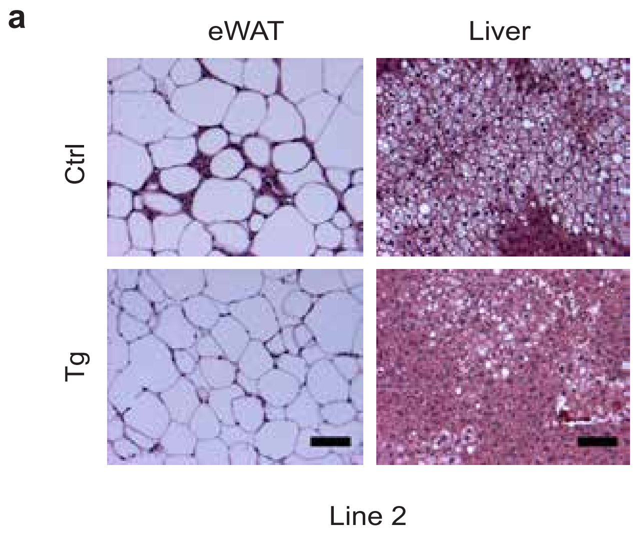

Supplementary Figure 7. Histology analysis in adipose depots in transgenic and control mice from Line 2 (a) Histologic analysis of eWAT and liver of from transgenic (Tg) and control (Ctrl) mice after 16 weeks of HFD (Line 2). Scale bar represents 100 μm.

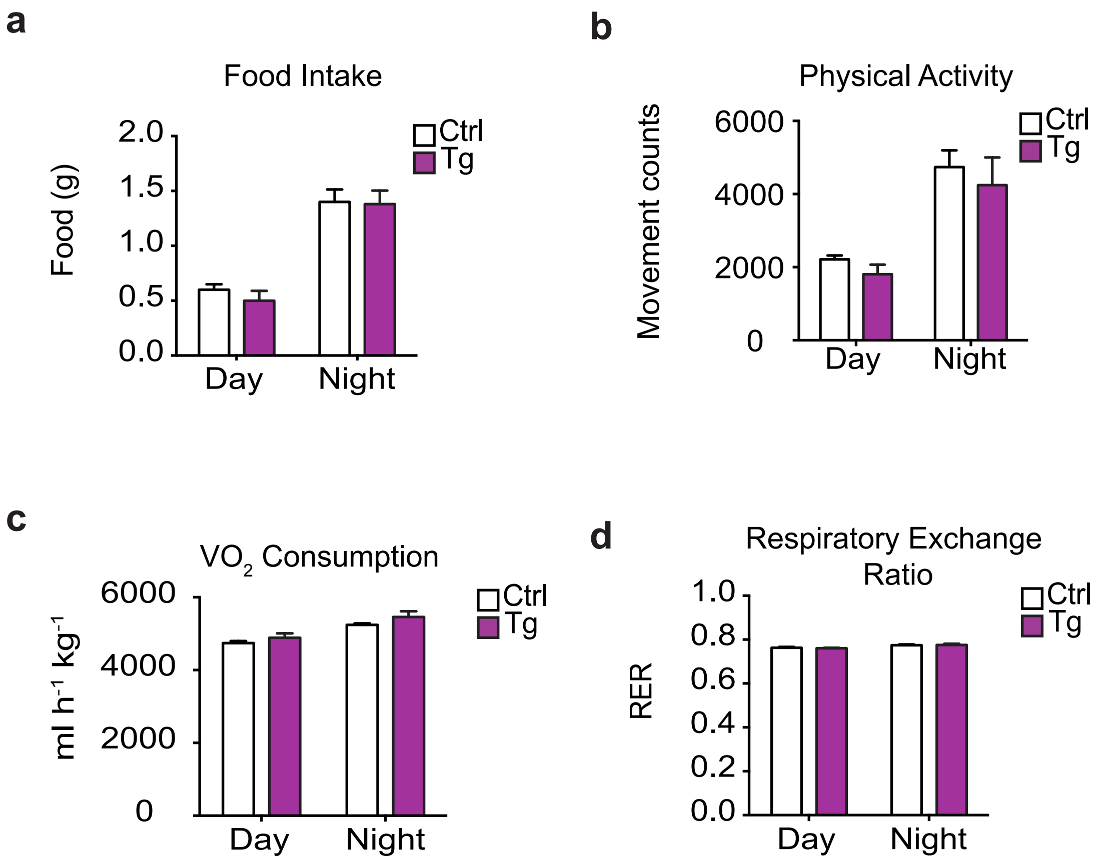

Supplementary Figure 8. Metabolic profiling in *Tnmd* transgenic mice. Metabolic cage analysis was performed in male mice that had been fed with HFD for 12 weeks. **(a)** Avarage food intake per mouse during the day (7am-7pm) and night (7pm-7am) over 3 days (mean±SEM; n=8 (control), n=5 (transgenic)). **(b)** Physical activity was calculated as avarage of total movements per mouse during the day (7am-7pm) and night (7pm-7am) over 3 days (mean±SEM; n=8 (control), n=5 (transgenic)). Avarage **(c)** VO_2 consumption and **(d)** Respiratory Exchange Ratio during the day (7am-7pm) and night (7pm-7am) over 3 days (mean±SEM; n=8 (control), n=5 (transgenic)).

a

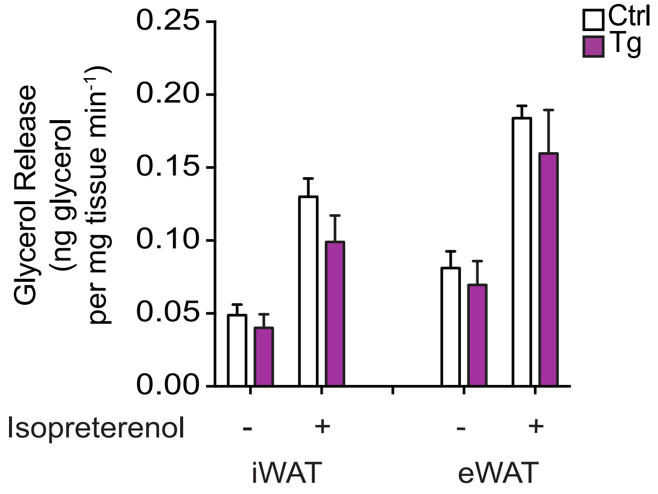

Supplementary Figure 9. Ex vivo lipolysis in adipose tissue explants (a) 6-8 weeks old mice were fed with chow diet for 12 weeks. Adipose tissue explants were isolated and lipolysis was stimulated with 10 μM isopreterenol for 2 hours (mean±SEM; n=6 (control), n=7 (transgenic), Student’s t-test).

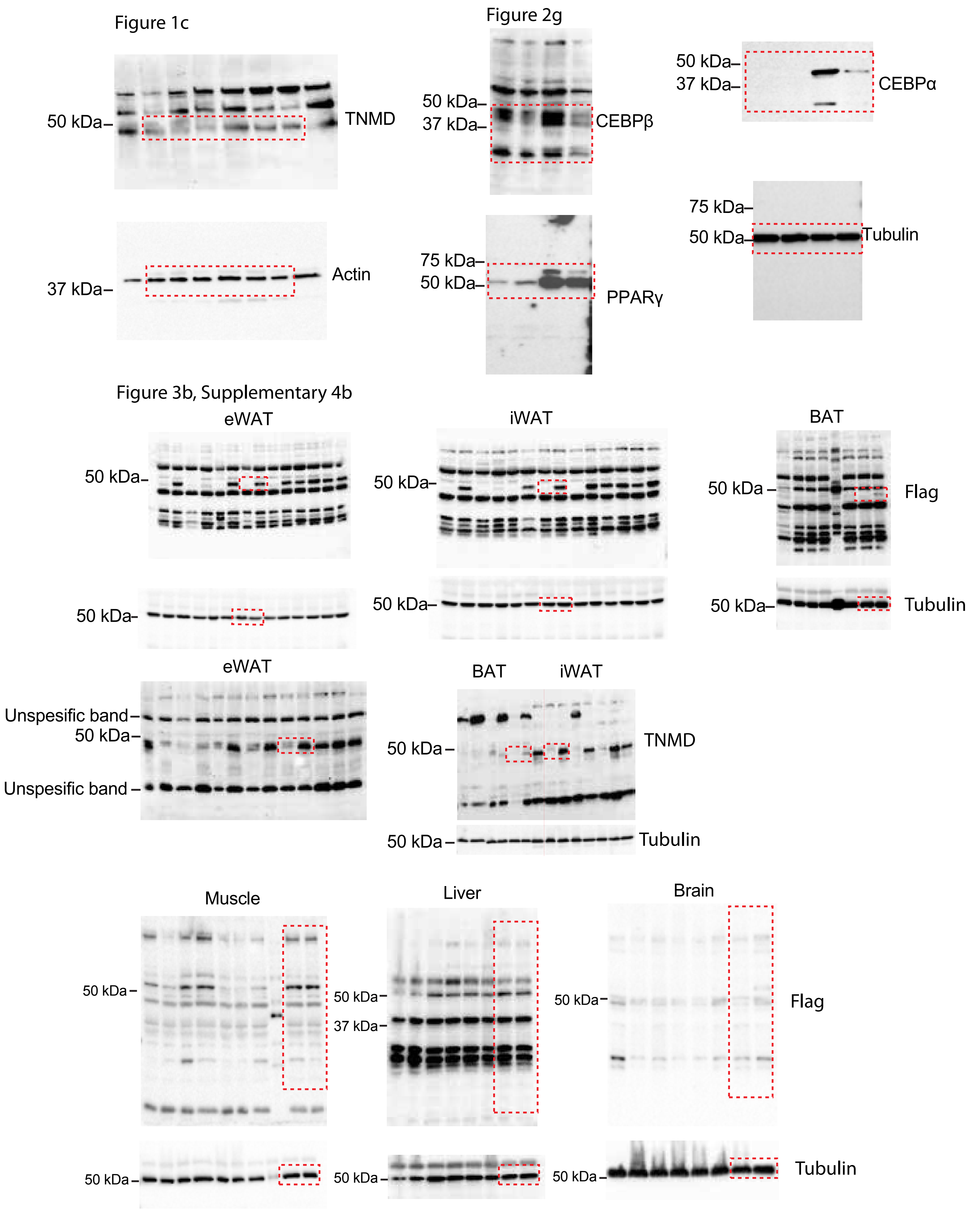

Supplementary Figure 10. Uncropped gel scans for Figure 1c, 2g, Figure 3b and Supplementary Figure 4b.

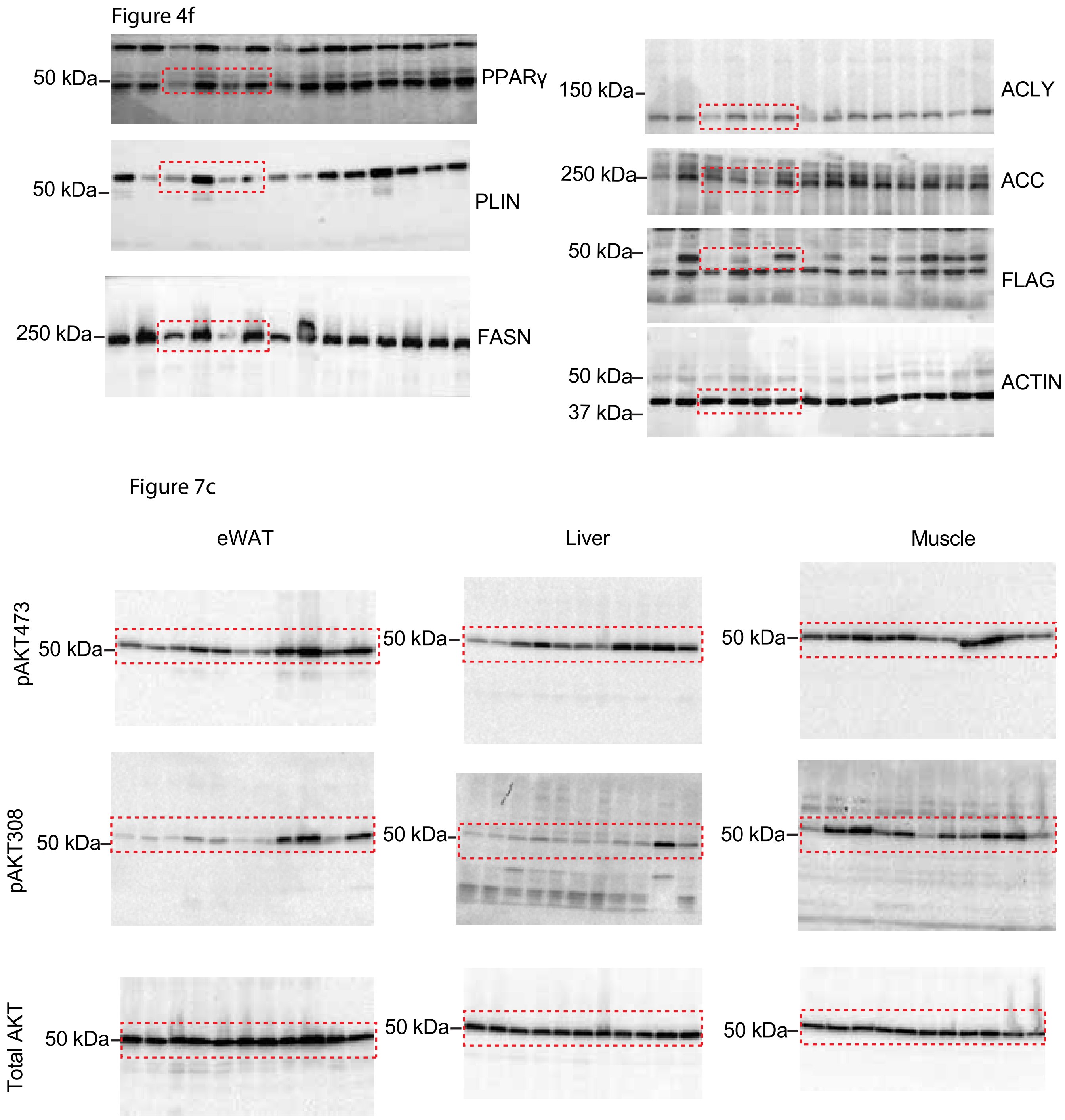

Supplementary Figure 11. Uncropped gel scans for Figure 4f and Figure 7c

	Insulin Sensitive (n=8) (Mean±SE)	Insulin Resistant (n=6) (Mean±SE)	*P*
**Age**	37 (±1.57)	44 (±3.72)	0.1060
**Weight**	295.875 (±14.89)	317.17 (±22.38)	0.4257
**BMI**	48.25 (±1.04)	50 (±3.34)	0.5829
**EBW**	167.25 (±11.15)	182.67 (±21.05)	0.5006
**Fasting glucose (mg/dl)**	88.125 (±3.28)	104.83(±5.20)	0.0145
**Fasting insulin (mIU/l)**	9.375 (±1.54)	24 (±3.72)	0.0017
**HOMA-IR (mmol/l)**	1.375 (±0.22)	3.57 (±0.53)	0.0013
**%B Beta cell sensitivity**	125.4 (±18.47)	174.12 (±25.89)	0.1405

Supplementary Table 1. Clinical characteristics of subjects selected for human Affymetrix GeneChip analysis.

BMI: Body-Mass Index

EBW: Excess body weight

HOMA-IR: homeostasis model assessment of insulin resistance

Human Primers		
**Gene**	**Forward**	**Reverse**
TNMD	ATTCAGAAGCGGAAATGGCACTGA	TAGGCTTTTCTGCTGGGACCCAA
C/EBPA	CAAGAACAGCAACGAGTACCG	GTCACTGGTCAGCTCCAGCAC
PPARG2	CTCCTATTGACCCAGAAAGCGA	TGCCATGAGGGAGTTGGAAG
PLIN1	GACCTCCCTGAGCAGGAGAAT	GTGGGCTTCCTTAGTGCTGG
KLF4	GATGAACTGACCAGGCACTA	TCGGGAAGACAGTGTGAAAA
SPP1	CTCCATTGACTCGAACGACTC	CAGGTCTGCGAAACTTCTTAGAT
ADIPOQ	TCTGCCTTCCGCAGTGTAGG	GGTGTGGCTTGGGGATACGA
RPLP0	CAGATTGGCTACCCAACTGTT	GGGAAGGTGTAATCCGTCTCC

Mouse Primers		
**Gene**	**Forward**	**Reverse**
Tnmd	AATGGGTGGTCCCGCAAGTG	ACAGACACGGCGGCAGTAAC
C/ebpa	CAAGAACAGCAACGAGTACCG	GTCACTGGTCAGCTCCAGCAC
C/ebpb	GCAAGAGCCGCGACAAG	GGCTCGGGCAGCTGCTT
Pparg2	TCGCTGATGCACTGCCTATG	GAGAGGTCCACAGAGCTGATT
Cidec	ATCAGAACAGCGCAAGAAGA	CAGCTTGTACAGGTCGAAGG
Lept	GAGACCCCTGTGTCGGTTC	CTGCGTGTGTGAAATGTCATTG
Adipoq	TGTTCCTCTTAATCCTGCCCA	CCAACCTGCACAAGTTCCCTT
Glut4	GTGACTGGAACACTGGTCCTA	CCAGCCACGTTGCATTGTAG
Plin1	CTGTGTGCAATGCCTATGAGA	CTGGAGGGTATTGAAGAGCCG
Srebp1c	GGCCCGGGAAGTCACTGT	GGAGCCATGGATTGCACATT
Fasn	GGAGGTGGTGATAGCCGGTAT	TGGGTAATCCATAGAGCCCAG
Acly	ACCCTTTCACTGGGGATCACA	GACAGGGATCAGGATTTCCTTG
Acc1	TGTACAAGCAGTGTGGGCTGGCT	CCACATGGCCTGGCTTGGAGGG
Cd68	CCATCCTTCACGATGACACCT	GGCAGGGTTATGAGTGACAGTT
Ccl2	GCTGGAGAGCTACAAGAGGATCACC	TCCTTCTTGGGGTCAGCACAGAC
Il-6	TAGTCCTTCCTACCCCAATTTCC	TTGGTCCTTAGCCACTCCTTC
Tnfa	CCCTCACACTCAGATCATCTTCT	GTCACGACGTGGGCTACAG
Ucp1	ACTGCCACACCTCCAGTCATT	CTTTGCCTCACTCAGGATTGG
Prdm16	CCAAGGCAAGGGCGAAGA	AGTCTGGTGGGATTGGAATGT
Plin2	GACCTTGTGTCCTCCGCTTAT	CAACCGCAATTTGTGGCTC
Mmp2	AACACTGAAGATCTTGCTCTGAGAT	TTGAGAGACTGAGACAGGGAGTC
Mmp3	TGATGAACGATGGACAGAGG	GAGAGATGGAAACGGGACAA
Mmp9	ACCACATCGAACTTCGA	CGACCATACAGATACTG
Mmp12	CTGGACAACTCAACTCT	AGAGGAGTCACATCACT
Mmp14	CAGTATGGCTACCTACCTCCAG	GCCTTGCCTGTCACTTGTAAA
Fn1	ATGTGGACCCCTCCTGATAGT	GCCCAGTGATTTCAGCAAAGG
Smad2	ATGTCGTCCATCTTGCCATTC	AACCGTCCTGTTTTCTTTAGCTT
Smad3	CACGCAGAACGTGAACACC	GGCAGTAGATAACGTGAGGGA
Cola1a1	GCTCCTCTTAGGGGCCACT	CCACGTCTCACCATTGGGG
Col5a1	CTTCGCCGCTACTCCTGTTC	CCCTGAGGGCAAATTGTGAAAA
Col6a1	AACAGGAATAGGAAATGTGACCC	ACACCACGGATAGGTTAGGGG
Col6a3	CAGAACCATTGTTTCTCACT	AGGACTACACATCTTTTCAC
Tgfb1	ATTCCTGGCGTTACCTTGG	AGCCCTGTATTCCGTCTCCT
Vwf	CTTCTGTACGCCTCAGCTATG	GCCGTTGTAATTCCCACACAAG
Cd31	ACGCTGGTGCTCTATGCAAG	TCAGTTGCTGCCCATTCATCA
Cdh5	CACTGCTTTGGGAGCCTTC	GGGGCAGCGATTCATTTTTCT
Thbs1	GGGGAGATAACGGTGTGTTTG	CGGGGATCAGGTTGGCATT
36b4	TCCAGGCTTTGGGCATCA	CTTTATCAGCTGCACATCACTCAGA

Supplementary Table 2. Primer sequences used in qRT-PCR analysis.

**Human *TNMD***	
Sequence 1	CGUCGAGGCAACCGCUAUU
Sequence 2	CCGCGUCUGUGAACCUUUA
Sequence 3	GAGAGAGGUUAUUGUUGUA
Sequence 4	GAAGAAGAUUUACAUGGAA
Mouse TNMD	
Sequence 1	GCGAUAAUGUGACCAUGUA
Sequence 2	GAAGAUUUGUGGACUAGUG
Sequence 3	GAUCAAUCCCACUCUAAUA
Sequence 4	GGCCUUAACUCUAAUUGUC

Supplementary Table 3. Target sequences for individual siRNA for smart pool for human and mouse *TNMD.*
